# Mechanistic Rationale and Clinical Efficacy of Hyperbaric Oxygen Therapy in Chronic Neuropathic Pain: An Evidence-Based Narrative Review

**DOI:** 10.1155/2021/8817504

**Published:** 2021-04-22

**Authors:** Simone Schiavo, Julian DeBacker, Carine Djaiani, Anuj Bhatia, Marina Englesakis, Rita Katznelson

**Affiliations:** ^1^Hyperbaric Medicine Unit, Department of Anesthesiology and Pain Medicine, Toronto General Hospital, University Health Network, University of Toronto, Toronto, Ontario, Canada; ^2^Department of Anesthesiology and Pain Medicine, Toronto Western Hospital, University Health Network, University of Toronto, Toronto, Ontario, Canada; ^3^Library and Information Services, Toronto General Hospital, University Health Network, Toronto, Ontario, Canada

## Abstract

**Background:**

Chronic neuropathic pain is a condition affecting an increasing proportion of the general population and its management requires a comprehensive, multidisciplinary program. A growing body of evidence supports the use of hyperbaric oxygen therapy (HBOT) in several chronic neuropathic pain conditions; however, its role and efficacy remain unclear.

**Purpose:**

To summarize current evidence for the mechanistic rationale of HBOT in chronic neuropathic pain conditions and to evaluate its clinical efficacy.

**Methods:**

This narrative review was conducted after searching the following databases (Medline, Embase, Cochrane, PsycINFO, the Web of Science, Scopus, ClinicalTrials. gov, WHO ICTRP, and ProQuest Digital Dissertation) from January 1946 to March 2020. Articles published in English that involved either animal or human studies with acute or chronic neuropathic pain evaluating any HBOT-related intervention were included.

**Results:**

A total of 2971 citations were identified. A total of 29 studies were included in this review. The mechanisms of action for HBOT use in neuropathic conditions included the primary effects of hyperoxia and edema resolution, as well as the secondary effects pertinent to the production of oxygen and nitrogen reactive species (serving as pain signaling molecules), nitric oxide-dependent release of opioid peptides, and reduction of inflammatory mediators. A robust evidence for HBOT use in the clinical setting was associated with chronic regional pain syndrome and chronic primary bladder pain syndrome. Some evidence supported its use for chronic secondary (peripheral) neuropathic pain including radiation-induced plexus neuropathies, postherpetic neuralgia, and trigeminal neuralgia.

**Conclusions:**

HBOT has been shown to have antinociceptive and analgesic effects in animal models of inflammatory, neuropathic, and chronic pain. Human studies demonstrated beneficial effects of HBOT in improving clinical outcomes such as pain scores, pain-related symptoms, and quality of life. A systematic methodology of HBOT application is necessary to confirm its safety and efficacy.

## 1. Introduction

Chronic neuropathic pain is a silent, global health epidemic requiring a novel approach to improve the treatment methodologies. It is estimated that 10–30% of adults live with a chronic pain condition and more than half of these patients report continuous moderate to severe pain and reduced quality of life resulting in job loss or reduced efficacy at workplace [1, 2].

Neuropathic pain, as defined by the International Association for the Study of Pain (IASP) Neuropathic Pain Special Interest Group, is “pain arising as a direct consequence of a lesion or disease affecting the somatosensory system” [3]. Controversy in understanding chronic pain as a symptom or a disease has led to a new classification for chronic pain disorders [4], reclassifying some neuropathic pain conditions as chronic primary pain disorders [5] (Table 1) and others as chronic secondary (central or peripheral) neuropathic pain disorders [3] (Table 2). Primary chronic pain disorders differ from the secondary ones in that they are independent of identified biological or psychological conditions.

Currently, the first-line management strategy for chronic neuropathic pain is based on multifaceted approach including tricyclic antidepressants, serotonin norepinephrine reuptake inhibitors, and gabapentinoids, as well as topical and transdermal substances. The second-line therapy consists of a combination of first-line medications with tramadol or tapentadol. Serotonin-specific reuptake inhibitors/anticonvulsants/N-methyl-D-aspartate receptor antagonists and interventional therapies are suggested as a third-line option. Neurostimulation is a fourth-line treatment. Low-dose opioids (no greater than 90 morphine equivalent units) are the fifth-line approach. Finally, a targeted drug delivery is considered as the last option for patients who are resistant to all other therapeutic interventions [6].

Given that the current pharmacological and interventional therapies are not devoid of significant adverse events, there is an urgent need for new, effective, noninvasive therapies.

A growing body of evidence supports the use of hyperbaric oxygen therapy (HBOT) in a number of neuropathic pain conditions with persistent efficacy and minimal adverse events.

HBOT was initially developed to treat decompression sickness, a complication of deep sea diving. In decompression sickness, a diver has surfaced before dissolved gases have time to equilibrate, producing space-occupying embolic nitrogen bubbles in the tissues and vasculature. HBOT provides patients with 100% oxygen at pressures two to three times greater than atmospheric pressure. According to Boyle's law, the increase of hydrostatic pressure in the hyperbaric chamber elevates the partial pressure of gases and causes a reduction in the volume of the gas-filled spaces, allowing them to dissolve into solution [7]. HBOT also increases partial pressure of oxygen in the alveoli and results in a corresponding increase in the amount of dissolved oxygen carried in blood. During HBOT, the arterial partial pressure of oxygen (PaO_2_) may reach up to 1,900–2,100 mmHg, with dissolved oxygen (O_2_) content increasing from 0.3 to 6.8 ml O_2_/100 ml of blood. This translates to a significant increase in partial pressure of O_2_ in tissues with compromised circulation [8]. A list of current conditions approved for treatment with HBOT by the Undersea & Hyperbaric Medicine Society is reflected in Table 3 [9]. For the majority of indications, the HBOT treatments are administered daily for 90–120 minutes for a total of 20–40 consecutive sessions. HBOT is considered a safe, noninvasive therapy with very few side effects (Table 4) [10]. The only absolute contraindication for HBOT is an untreated pneumothorax.

**Figure 1:**
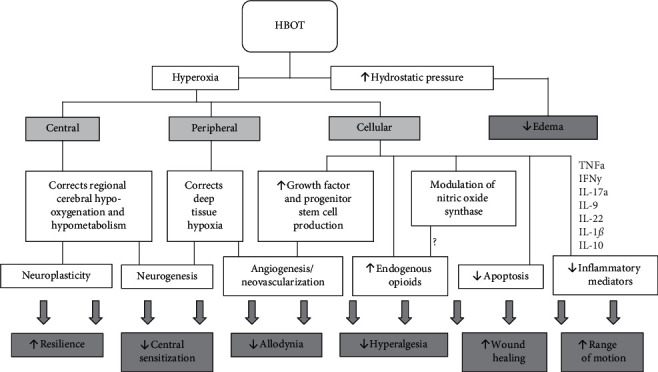
HBOT mechanisms and effect on chronic neuropathic pain disorders. HBOT: hyperbaric oxygen therapy; ROS: reactive oxygen species; RNS: reactive nitrogen species; TNF*α*: tumor necrosis factor-alpha; IFN*γ*: interferon-gamma; IL: interleukin.

HBOT is an effective treatment of neuropathic pain conditions due to its primary and secondary effects (Figure 1). The primary effects relate to significant increase in partial pressure of tissue O_2_ (hyperoxia) [8]. Hyperoxia serves as a potential mechanism for treatment of pain conditions with evidence of deep tissue hypoxia, such as in the chronic regional pain syndrome (CRPS) [11, 12]. Increased hydrostatic pressure is another primary effect of HBOT. It induces arteriolar vasoconstriction that reduces formation of tissue edema, without compromising the effect of hyperoxia [13].

The secondary effects of HBOT involve the production of reactive oxygen species (ROS) and reactive nitrogen species (RNS). These reactive species serve as signaling molecules implicated in pain perception, wound healing, angiogenesis, neovascularization, leukocyte function, growth factor, and progenitor stem cell release, as well as tissue homeostasis [8].

Another secondary effect of HBOT is related to reduction in inflammatory mediators.

The objective of this review was to summarize evidence for the mechanistic rationale of HBOT in chronic neuropathic pain conditions and to evaluate its clinical efficacy in this patient population.

## 2. Methods

The methodology used was consistent with the PRISMA Extension for Scoping Reviews Checklist (PRISMA-ScR) [14]. The following databases were searched from January 1946 to March 21, 2020, via the Ovid search interface: Medline, Medline In-Process/ePubs, Embase, Cochrane Central Register of Controlled Trials, Cochrane Database of Systematic Reviews, and PsycINFO. The Web of Science (Clarivate) and Scopus databases, ClinicalTrials.gov (NIH), WHO ICTRP, and ProQuest Digital Dissertation were also included in our search strategy. Search strategy concept blocks were built on the topics of Hyperbaric Oxygen Therapy and Neuropathic Pain, Hyperbaric Oxygen Therapy and Chronic Primary Bladder Pain Syndrome, and Hyperbaric Oxygen Therapy and Complex Regional Pain Syndrome (CRPS). The Medline search strategy is provided in detail in Supplementary Material. We included all articles published or translated in English that involved either animal or human studies with acute or chronic neuropathic pain evaluating any HBOT-related intervention. Study outcomes had to include an assessment of any pain-related or physiologic endpoints, either objective (e.g., cytokine blood levels, imaging, and tissue oxygenation) or subjective (e.g., questionnaires). In order to limit the scope of the review, we excluded studies examining chronic primary headache or orofacial pain (such as migraine, cluster, and tension headaches) and, within the secondary neuropathic pain, the painful diabetic neuropathy, because the abundant literature on these topics over the last four decades would deserve a separate review and is out of the scope of the current analysis. Study selection was performed by two reviewers (SS and JDB) using inclusion and exclusion criteria. The third reviewer (RK) was available to solve any disagreements related to selection process.

## 3. Results

**Figure 2:**
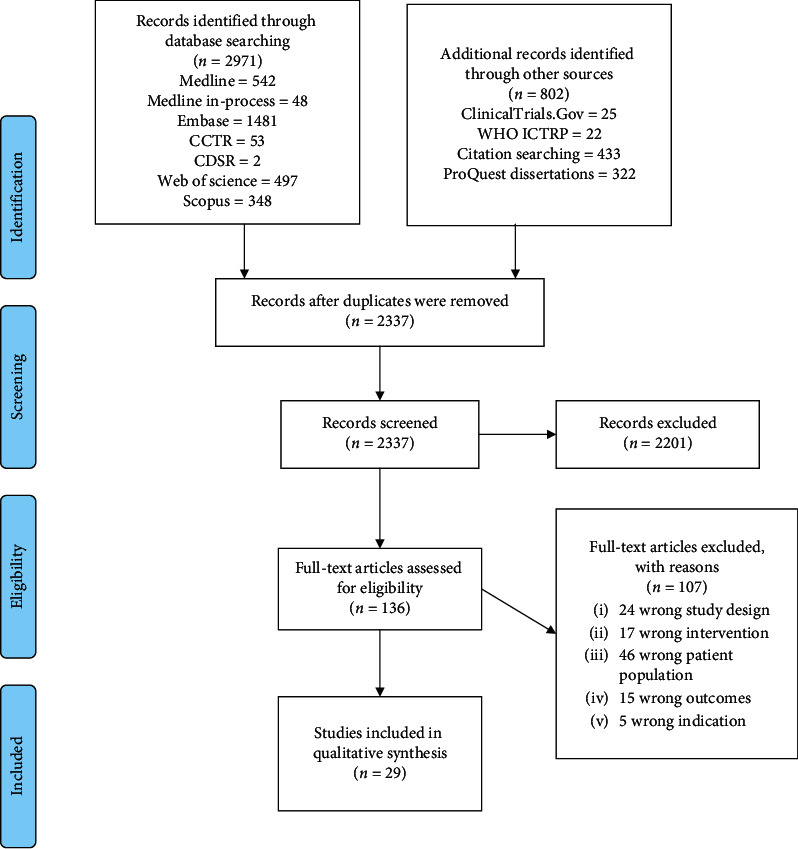
PRISMA flow diagram.

A total of 2971 records were identified through database searching, while 505 references were identified with the search strategy. Furthermore, there were 802 additional records through other sources, including 433 Citation Searching and 322 dissertations. 1436 duplicates were identified and removed by the specific Covidence software tool. After 2337 records were screened, 2201 were excluded. A total of 136 references met the inclusion criteria. 107 records were excluded. Finally, 29 studies were included in the qualitative synthesis (Figure 2, PRISMA flow diagram).

### 3.1. HBOT in Animal Models of Neuropathic Pain

Animal models for both neuropathic and inflammatory pain conditions demonstrate the antiallodynic and antinociceptive effects of HBOT through a wide range of treatment regimens.

The most common animal model for neuropathic pain is a chronic constriction injury (CCI) to the sciatic nerve. In this model investigators tie four ligatures around the sciatic nerve to create intraneural edema and ischemia, neuronal apoptosis, and Wallerian degeneration [15]. Shortly after CCI, rats exhibit neuropathic pain behaviors including mechanical, chemical, and thermal hyperalgesia and allodynia persisting for up to two months [15]. Other pain-related characteristics including appetite suppression, nocifensive behaviors (scratching, biting, self-mutilation), and increased anxiety- and depression-like behaviors following CCI implicate cognitive and affective sequalae of this neuropathic pain model [16].  Table 5 summarizes 9 animal studies of neuropathic pain that all demonstrate the efficacy of HBOT, across a wide spectrum of treatment regimens, in reducing the thermal and mechanical hyperalgesia and allodynia induced by experimental nerve injury [17–25]. While some of these studies demonstrate sustained (up to one month) antiallodynic and antinociceptive effects with only one HBOT session, the majority of studies suggest a dose-response with sustained therapeutic benefit after multiple consecutive HBOT sessions. Suggested mechanisms for the therapeutic effects of HBOT in this animal model include inhibition or suppression of CCI-induced inflammation [17, 18, 22], neuronal apoptosis [21, 25], and increased expression of endogenous opioids [20]. CCI-induced expression of nitric oxide synthase isoforms, which are involved in the modulation of periphery and central nociceptive pathways, are also attenuated following HBOT [23, 24].

Animal studies have also suggested potential benefits of HBOT in chemotherapy-induced neuropathic pain. In one study [26], 7-day exposure to paclitaxel induced mechanical and cold allodynia in rats. After 1–4 treatments of HBOT, mechanical allodynia was completely reversed, while cold allodynia was not reliably reduced. Another study [27] examined the effects of HBOT as a treatment and prophylaxis in cisplatin-induced peripheral neuropathy. After twice-weekly intraperitoneal injections of cisplatin over a period of four weeks, mechanical allodynia was observed in rats following the first week of cisplatin exposure and persisting throughout the three following weeks. A 7-day HBOT treatment course following 4 weeks of cisplatin exposure did not improve the mechanical nociceptive threshold; however the 7-day HBOT treatment course before the 4 weeks of cisplatin exposure did significantly improve mechanical allodynia. In this “preconditioning” group, the antinociceptive benefit of HBOT was attributed to decreased apoptosis and inflammation. Immunohistochemical analysis of the sciatic nerve and associated ganglia demonstrated decreased upregulation of caspase-3 expression (proapoptotic mediator) and an attenuated expression of TNF-*α* and inducible NOS expression when compared to the control group. In combination with the CCI-model of neuropathic pain, these animal studies further validated some of the proposed antiallodynic and antinociceptive mechanisms of HBOT.

Hyperoxygenation serves as a potential mechanism for treatment of neuropathic pain conditions that show evidence of deep tissue hypoxia, such as in chronic regional pain syndrome (CRPS) [11, 12]. The deep tissue hypoxia hypothesis has been tested in the animal model of ischemia and reperfusion related chronic pain. Known as “chronic postischemia pain,” a tourniquet is placed around a rat's ankle for 3 hours under general anesthesia and then released. After reperfusion, the hind paw exhibits an initial phase of hyperemia and edema lasting for 2–12 hours, followed by neuropathic pain (mechano-hyperalgesia, mechano-allodynia, and cold allodynia) that lasts for at least one month [28]. Using this ischemia-reperfusion model, Coderre et al. [11] discovered microvascular injury in the capillaries of deep muscles and nerves. Their findings implicated deep tissue and endoneurial ischemia and inflammation in the activation of both muscle nociceptors and ectopic sensory afferent axons [11]. The efficacy of HBOT, however, has not yet been tested in this animal model. The deep tissue hypoxia hypothesis for chronic pain also extends to the central nervous system. This is because regional, cerebral hypoperfusion and hypometabolism have been associated with chronic pain conditions, such as fibromyalgia and CRPS [29–31]. HBOT appears to correct or rectify these regional differences in brain perfusion and metabolism, inducing changes that are associated with reduction in pain symptoms and improved quality of life [32].

Increased hydrostatic pressure is another primary effect of HBOT. HBOT induces arteriolar vasoconstriction by subsequently reducing tissue edema formation, without compromising the supernormal tissue pO_2_ [13]. This mechanism may contribute to a reduction in symptoms in patients with chronic pain conditions where tissue edema and inflammation are the primary components [33]. An animal model of inflammatory pain and edema has tested this theory. A subcutaneous injection of 1% carrageenan substance into rats' hind paws induced both mechanical hyperalgesia and edema providing a close clinical surrogate to inflammatory pain in humans [28]. Mechanical hyperalgesia or hypersensitivity was measured using the “mechanical paw withdrawal threshold (MPWT),” whereby von Frey filaments of increasing diameter (increasing force) poked the animals' hind paws through a mesh cage. Compared to sham group, HBOT-treated rats exhibited decreased mechanical hyperalgesia/hypersensitivity (a higher MPWT) almost immediately following treatment. There was also evidence for the decreased edema formation after HBOT; however, it lagged slightly behind the antinociceptive effect [28].

Secondary effects of HBOT that may underlie the treatment of chronic pain disorders involve the production of ROS and RNS. In animal models for neuropathic pain, the therapeutic effect for HBOT involves a reduction or attenuation of CCI-induced nitric oxide synthase expression [23, 24]. However, in experimental models of abdominal pain the opposite is shown [29, 31].

Zelinski et al. [29] sought to elucidate the mechanism underlying the antinociceptive effect of HBOT in their animal model of chronic pain. Following intraperitoneal injection of 0.6% glacial acetic acid, mice exhibited abdominal constrictions (lengthwise stretches of the torso with concave arching of the back), presumed to be a pain response that was quantifiable (number of constrictions). HBOT showed a profound reduction in abdominal constrictions compared to those in the untreated mice [29].

In search of a mechanistic explanation for the antinociceptive effect of HBOT in this model the group hypothesized a neural nitric oxide- (NO-) dependent mechanism as it was known that HBOT increased cerebral blood flow and RNS production through neural NO synthase (nNOS) activation in the cerebral cortex [30]. They discovered that central administration (intracerebroventricular or intrathecal) of NOS enzyme inhibitors and antisense oligodeoxynucleotides against nNOS attenuated HBOT-mediated analgesia in mice. A similar attenuation was noted in nNOS knockout mice and with naltrexone (opioid antagonist) administration [29, 31]. While both neuropathic and abdominal/inflammatory models of pain demonstrate the release of endogenous opioids as a therapeutic mechanism, the pro- or antinociceptive role of NOS and RNS remains controversial [34] and may vary at different locations within the central and peripheral nervous system as well as with different etiology of chronic pain.

### 3.2. HBOT in Human Studies of Neuropathic Pain

#### 3.2.1. HBOT in Complex Regional Pain Syndrome

A total of 5 case reports and one RCT described effects of HBOT in CRPS patients (Tables 6 and 7).

CRPS is a chronic primary pain syndrome characterized by spontaneous and/or evoked pain disproportionate to the typical course of pain produced from a similar inciting event. The pain distribution is not limited to specific nerve territory or dermatome and often has a distal spread. It can be accompanied by sensory (hyperalgesia/allodynia), vasomotor, sudomotor/edema, and motor/trophic changes [47]. CRPS is caused by a multifactorial process involving both peripheral and central mechanisms as well as sensitization. Inflammatory and immune-related factors, altered sympathetic nervous system function, ischemic reperfusion injury, and oxidative stress are among the many possible mechanisms implicated in CRPS [48, 49]. In addition to anti-inflammatory activity [17, 28], four possible mechanisms are proposed to account for the improvement in symptoms and quality of life that is seen following HBOT in CRPS patients.

#### 3.2.2. HBOT and Reduction of Deep Tissue Hypoxia

Bellingham et al. [12] demonstrated that deep tissue oxygen saturation (StO_2_) was significantly lower in the affected limb of patients with CRPS compared to either the nonaffected limb or healthy volunteers. Given that HBOT significantly increases StO_2_ [50], the “deep tissue hypoxia” theory is an attractive mechanism for the proposed benefit of HBOT, although the changes in StO_2_ before and after HBOT have not been documented in patients with CRPS. Improved tissue oxygenation was the suspected mechanism in the first report of HBOT benefit in a CRPS patient. A case study from 1995 [35] reported a 44-year-old woman with CRPS treated with emergent HBOT for acute smoke inhalation for 46 minutes at 2.8 ATA. 15 minutes after the start of the therapy the patient “reported relief of pain in her foot” that appeared “less cyanotic and warmer to the touch.” The foot color remained pink for 8 hours and painless for 18 hours after the first HBOT session. The patient was offered a second 90-minute HBOT session at 2.0 ATA with mild symptom improvement (the foot remained pink for 1 hour and painless for 2 hours after HBOT) and one additional session at 2.4 ATA, with marked and long-lasting relief (the foot was pink and painless for 30 hours after HBOT). While no precise methodology for assessment was reported, the clinical findings were thought to be related to improved perfusion and oxygenation with the corresponding improvement in pain control.

#### 3.2.3. HBOT and Reduction of Tissue Edema

A second potential mechanism of HBOT is related to decreased tissue edema, which is a common feature of CRPS. It is well described that HBOT causes vasoconstriction and decreases edema [13, 33]. It is suggested that the hyperoxic environment leads to increased oxidation of NO radicals produced by the endothelium and a loss of the vasorelaxant effect [51], alterations in other vasodilator compounds (i.e., prostaglandins) [52], and stimulation of central vasoregulation via the sympathetic nervous system [53]. Indeed, an RCT of patients with CRPS [33] demonstrated improved range of motion and decreased edema following HBOT. In this double-blind RCT, 71 patients with posttraumatic wrist CRPS were randomized to a treatment group (37 patients), receiving 15 HBOT sessions, 90 minutes each at 2.4 ATA, or a control group (34 patients) that received 15 placebo sessions in the hyperbaric chamber, 90-minute each breathing normal air at 2.4 ATA. In addition to HBOT, all patients received paracetamol 500 mg three times a day but did not receive any physical therapy during the study. Assessments included evaluation of visual analogue pain scores, range of motion (goniometric assessment of wrist flexion and extension), and edema (wrist circumference) at baseline, at the end of the 15 HBOT sessions, and at 45-day follow-up. The HBOT group reported significantly lower pain scores, improved range of motion, and decreased edema, both at the end of HBOT and at the follow-up sessions, while the control group did not have any improvement. The authors concluded that HBOT was effective in decreasing pain and swelling and increasing wrist range of motion in patients with CRPS. Given that the sham control was also exposed to higher pressure (breathing air at 2.4 ATA), this study implicates the therapeutic benefits of hyperoxia rather than just increased atmospheric pressure environment, although it should be noted that even the control group experienced higher levels of oxygenation at 2.4 ATA.

#### 3.2.4. Role of HBOT in Acute and Chronic CRPS

Another possible mechanism for HBOT in CRPS is in preventing progression of the disease from the early acute and dystrophic stages to the irreversible/atrophic stage that is characterized by stiffness and flexion contractures. A critique of the previous RCT [33] was that the population studied were young and otherwise healthy soldiers receiving HBOT within 1.5 months of the original injury. It was postulated that because HBOT stimulates the activity of osteoblasts and decreases formation of fibrosis [54], it interrupts the basic vicious cycle of CRPS pathophysiology.

Williams et al. [36] described a case report of a 48-year-old diabetic man who developed complications after elective subtalar arthrodesis for chronic ankle instability. Following two irrigations and debridement for infection, the patient received 19 sessions (90 min at 2.2 ATA) of HBOT for wound healing enhancement, starting 3 weeks after the first surgery. One month after HBOT the wound was completely healed, but the patient started to develop neuropathic pain and other symptoms pertinent to CRPS. The authors concluded that HBOT, even if initiated early after a traumatic extremity injury, did not confer any protection against the possibility to developing CRPS.

Two case reports described potential benefits of HBOT in patients with chronic CRPS [37, 38]. A 41-year-old male with a 1-year history of left-foot CRPS following ankle fracture demonstrated less pain, decreased swelling, less allodynia, improvement in skin color, and a range of motion of the lower limb after 3 weeks of HBOT [37]. Furthermore, his mood and walking ability as well as interactions with other people and enjoyment of life markedly improved. Another case report [38] described a 50-year-old female with an 8-year complicated history of CRPS following the left fifth proximal phalanx fracture, in which multiple conventional and nonconventional therapies failed. The disease was partially controlled with a high dose of prednisone (85 mg/day). After 40 HBOT sessions, 90 minutes each at 2.4 ATA, the prednisone dose was reduced to 9 mg/day, with marked improvement in symptoms. Five months after the HBOT sessions, the patient had a mild flare of symptoms and received a second HBOT course of 33 sessions, 90 minutes each at 2.0 ATA, again with marked improvement, and further decrease in prednisone to 5 mg/day. The patients also experienced significant reduction of steroid related complications including diabetes, hypertension, dyslipidemia, insomnia, skin integrity, infections, and bruising. Even though these case reports are promising, it is paramount to acquire more robust evidence to advocate for HBOT as a standard treatment in patients with acute and chronic CRPS.

#### 3.2.5. Cerebral Targets for HBOT in CRPS

A fourth potential mechanism for HBOT benefit is through central or cerebral effects. A study by Barad et al. [55] discovered decreased gray matter changes in the limbic system (posterior mid-cingulate cortex, bilateral pregenual anterior cingulate cortex, and orbitofrontal cortex) and left posterior insula and increased gray matter in the dorsal putamen and hypothalamus that is involved in the processing of pain in patients with CRPS. Duration of illness and increased pain intensity were correlated with gray matter atrophy in the dorsolateral prefrontal cortex and gray matter hypertrophy in the hippocampus and amygdala. While brain changes have not been demonstrated in HBOT with CRPS patients, preliminary data from the fibromyalgia population demonstrate a rectification of imbalanced brain activity after treatments with HBOT [32, 56].

### 3.3. HBOT in Chronic Primary Bladder Pain Syndrome

The reports of HBOT in chronic primary bladder pain syndrome are reflected in Tables 6 and 7.

Also known as interstitial cystitis, bladder pain syndrome (BPS) is characterized by chronic pelvic pain or discomfort, associated with at least one urinary symptom, such as persistent urge to void, increased frequency, and nocturia [57, 58]. Under the ICD-11 classification BPS is considered as a chronic primary pain disorder [5]. Ophoven et al. [39, 40] sought to determine the feasibility and efficacy of HBOT in BPS based on observations of decreased pelvic pain in patients undergoing HBOT for radiation cystitis. Following a feasibility study [39], van Ophoven et al. conducted a randomized, sham controlled, double-blind trial [40] whereby 14 patients (experimental group) received 30 HBOT treatments at 2.4 ATA for 90 minutes and 6 patients (control group) received a sham treatment breathing room air at 1.3–1.4 ATA. Pain intensity measured by visual analogue scores (VAS) decreased significantly in the experimental group compared to the control group at 3-month follow-up. A prospective cohort study by Tanaka et al. [41] evaluated 11 patients with BPS symptoms who received 10–20 HBOT treatments for 60 minutes each at 2.0 ATA. Seven “responders” demonstrated a significant improvement in pain scores at 12-month follow-up. A pilot study by Wenzler et al. [42] evaluated 9 patients with interstitial cystitis receiving 30 HBOT sessions of 90 minutes at 2.2 ATA. Five “responders” had considerable reduction in VAS at the 12-month follow-up. Histological findings from patients with BPS demonstrated decreased microvascular density in the suburothelial plexus which was indicative of reduced edema and decreased vascular congestion. Consequently, HBOT-mediated neoangiogenesis and increased tissue oxygenation may serve as two proposed mechanisms for improved pain relief in patients with BPS.

### 3.4. HBOT in Chronic Secondary Neuropathic Pain

Among the chronic secondary pain syndromes, there is a growing body of evidence in support of HBOT's efficacy in neuropathic (peripheral) pain syndromes such as radiation- and chemotherapy-induced plexus neuropathies, postherpetic neuralgia (PHN), and trigeminal neuralgia (TN). HBOT has been also effective in managing secondary neuropathic conditions such as HIV-associated neuropathy [59], peripheral nerve injury [60, 61], and optic neuropathy [62–67].

#### 3.4.1. Radiation-Induced Plexus Neuropathies

Efficacy of HBOT in radiotherapy induced brachial and sacral plexus neuropathy has been evaluated in three clinical studies (Tables 6 and 7).

Pritchard et al. [43] reported the use of HBOT as an adjunctive therapy for radiation-induced brachial plexopathy (RIBP), an intractable neuropathic pain resulting in severe motor dysfunction [68]. 34 patients with RIBP were randomized to either experimental (100% O_2_ at 2.4 ATA) or control (41% O_2_ at 2.4 ATA) groups for 30 sessions of 90 minutes. Clinical and neurologic assessments including warm sensory threshold (measuring the function of small sensory fibers) and pain and quality of life questionnaires were conducted after the end of the HBOT treatment period and at 1- and 2-year follow-up. No differences in clinical outcomes either between or within the groups were found for up to 1-year follow-up. However, 2 patients in the HBOT group exhibited normalization of the warm sensory threshold, and 2 other patients experienced normalization of chronic arm lymphedema. While the authors concluded that “there is currently no basis for recommending HBOT as a proven treatment for RIBP,” it should be noted that statistical analysis was performed for just a single primary endpoint, “warm sensory threshold,” and no formal analysis was done for secondary endpoints, including pain. Additionally, while there was no “within-group” benefit, it should be noted that the control group was breathing 41% O_2_ at 2.4 ATA, which corresponds to approximately 100% oxygen at 1 ATA. Indeed, breathing 100% oxygen at sea level is considered a therapy by itself, since the increase in blood oxygen content could exert beneficial effects [69].

A case report by Videtic and Venkatesan [44] noted the beneficial effects of HBOT on sacral plexopathy causing severe pelvic pain in a 55-year-old woman. While the HBOT indication was for sacral osteoradionecrosis due to radiotherapy for bladder leiomyosarcoma, severe pain was a main symptom. Her pain was not responding to opioid analgesics, anti-inflammatories, dexamethasone, and amitriptyline. The patient received 30 sessions of HBOT at 2.5 ATA with a gradual but constant decrease on opioid requirement. 12-month follow-up consultation revealed no pain and no pain medications apart from minimal dose of amitriptyline. Another case report [45] described a brachial plexopathy in a 45-year-old man who underwent radiotherapy for neck carcinoma. He developed severe left shoulder/arm pain and decreased range of motion (ROM) after 15 months and RIBP diagnosis was confirmed by brachial plexus magnetic resonance imaging (MRI). He received dexamethasone for 2 weeks and underwent 30 HBOT sessions at 2.4 ATA, 120 minutes each. At 2-month and 13-month follow-up the patient regained full ROM in his left arm and reported no pain. These clinical findings were corroborated by a significant decrease of abnormal enhancement on follow-up MRI at 6 months. The authors hypothesized that the potential benefit could be related to longer HBOT sessions (120 minutes) as well as an early diagnosis and treatment. The results of these case studies suggest a potential benefit of HBOT for radiation-induced neuropathic pain and merit further investigations.

#### 3.4.2. Postherpetic Neuralgia

Postherpetic neuralgia (PHN) consists of a persistent irritation and intermittent neuropathic pain, usually associated with allodynia and itching [70], that persist after a primary herpes zoster infection. Peng et al. [46] randomized 68 patients who developed acute herpes zoster infection within 2 weeks to either intervention group (receiving HBOT 30 sessions, twice daily, 80 minutes each at 2.2 ATA, in addition to medical therapy) or control group, receiving optimal medical therapy (antiviral [acyclovir], nerve nutritive [mecobalamin], pain relief [tramadol], and antidepressant [nortriptyline]). Effectiveness of HBOT was assessed by objective measures including period of blister resolution, scar formation time and percentage of patients developing PHN, and subjective assessments with questionnaires reflecting pain and depression. Based on these assessments, all patients were categorized into three different “therapeutic efficacy” classes, namely, healed, improved, or ineffective. Overall, the calculated therapeutic efficacy in the HBOT group was significantly higher than in the control group (97 vs 81%). The HBOT group exhibited a significant reduction in persistent PHN development when compared to control group (11 vs 31%) and in scar formation time (11 vs 14 days). Pain and depression scores decreased in both groups; however, they were significantly lower in the HBOT group compared to controls. The authors concluded that “the combination of HBOT and conventional pharmacological therapy was more effective than pharmacological treatment alone.” A critical appraisal [71] highlighted that the study outcomes were measured when by natural history one would expect the infection to have resolved anyway (within 5 weeks period) and that the numerical differences in pain scores between the two groups were lacking clinical significance. It is clear that further studies with long-term follow-up are needed to determine the efficacy of HBOT in patients with chronic PHN.

#### 3.4.3. Trigeminal Neuralgia

Trigeminal neuralgia (TN) is defined as a neuropathic facial pain condition, characterized by unilateral paroxysmal pain, evoked by trigger factors [72, 73]. Under the new ICD-11, TN overlaps two different categories and is considered an example of “multiple parenting,” since it can be classified as both a chronic primary pain (subcategory of chronic primary headache/orofacial pain) and a chronic secondary peripheral neuropathic pain [4]. Multiple clinical studies of HBOT have been conducted in patients with chronic migraine and cluster headaches [74]; however, they are beyond the scope of this review. Nonetheless, TN plays a special role in both mechanistic rationale and clinical efficacy of HBOT in patients with neuropathic pain.

Many animal models for TN involve constriction, compression, or ligation of neural structures [18, 75, 76] to produce mechanical or thermal allodynia. These models are either identical or parallel to those used in elucidating the mechanism by which HBOT exerts antinociceptive effects in animals as discussed earlier in the “animal models” section (Table 5). Gu et al. [18] evaluated the effect of HBOT following chronic constriction injury of the sciatic nerve in rats and also conducted a trial in humans with idiopathic TN. In the animal model, they demonstrated that repetitive HBOT produced a rapid, dose-dependent, and long-lasting inhibition of thermal hyperalgesia and mechanical allodynia. In the clinical study, 42 patients with TN, concurrently treated with carbamazepine, were randomized to a treatment group (22 patients) receiving 10 sessions of HBOT for 70 minutes at 1.8 ATA or to a sham group (20 patients) receiving the same treatment in a hyperbaric chamber breathing room air at atmospheric pressure. Effectiveness of HBOT was assessed by carbamazepine dose reduction and VAS score changes at the 6-month follow-up. After 10 HBOT sessions, the treatment group had a significant decrease in required carbamazepine dose that lasted up to 90 days. Carbamazepine dose was also significantly lower when compared to the sham group for up to 60 days after HBOT. This was associated with significant reduction in VAS up to 6 months after HBOT.

## 4. Conclusions

There is a growing body of evidence suggesting the potential benefits and therapeutic impact of HBOT in different chronic neuropathic pain conditions. The current literature confirms a wide heterogeneity in the HBOT treatment modalities in patients with different chronic neuropathic pain presentations suggesting that appropriate dose-response curve specifics should be considered for each condition. HBOT has been shown to reduce pain scores and improve pain-related symptoms and quality of life. Future research should focus on the identification of a subset of patients with chronic pain who can benefit from HBOT.

## Figures and Tables

**Table 1:** Classification of chronic primary pain [4, 5].

Diagnostic entity	Subcategory

Chronic widespread pain	(i) Fibromyalgia
CRPS	(i) CRPS-1 (ii) CRPS-2
Chronic primary headache or orofacial pain	(i) Chronic migraine (ii) Chronic tension-type headache (iii) Trigeminal autonomic cephalalgias (iv) Chronic primary temporomandibular disorder pains (v) Burning mouth syndrome (vi) Chronic primary orofacial pain
Chronic primary visceral pain	(i) Chronic primary chest pain syndrome (ii) Chronic primary epigastric pain syndrome (iii) Irritable bowel syndrome (iv) Chronic primary abdominal pain syndrome (v) Chronic primary bladder pain syndrome (vi) Chronic primary pelvic pain syndrome
Chronic primary musculoskeletal pain	(i) Chronic primary low back pain (ii) Chronic primary cervical pain (iii) Chronic primary thoracic pain (iv) Chronic primary limb pain

CRPS: chronic regional pain syndrome. Underlined: conditions included in this review.

**Table 2:** Classification of chronic secondary pain syndromes [3, 4].

Diagnostic entity	Subcategory	Syndromes

C. cancer-related pain	(i) C. cancer pain	C. visceral cancer pain, C. bone cancer pain, C. neuropathic cancer pain
	(ii) C. postcancer treatment pain	Postcancer medicine pain (painful chemo-induced polyneuropathy)
		Postradiotherapy pain (C. painful radiation-induced neuropathy)
C. postsurgical or posttraumatic pain	(i) C. postsurgical	CP after amputation, CP after spinal surgery, CP after thoracotomy, CP after breast surgery, CP after herniotomy, CP after hysterectomy, CP after arthroplasty
	(ii) C. posttraumatic pain	CP after burns injury, CP after peripheral nerve injury (i.e., CRPS Type 2), CP after spinal cord injury, CP after brain injury, CP after whiplash injury, CP after musculoskeletal injury
C. neuropathic pain	(i) C. peripheral neuropathic pain	Trigeminal neuralgia, chronic neuropathic pain after peripheral nerve injury, painful polyneuropathy, postherpetic neuralgia, painful radiculopathy (including chemo- or radio-induced)
	(ii) C. central neuropathic pain	CCNP associated with spinal cord injury, CCNP associated with brain injury, C central poststroke pain, CCNP associated with multiple sclerosis
C. secondary headache or orofacial pain		CH or COP attributed to trauma or injury to the head or neck (e.g., CP after whiplash injury), CH or COP attributed to cranial or cervical vascular disorder, CH or COP attributed to nonvascular intracranial disorder, CH attributed to a substance or its withdrawal, CH or COP attributed to infection, CH attributed to disorders of homoeostasis or their nonpharmacological treatment, CH or COP attributed to disorder of the cranium, neck, eyes, ears, sinuses, salivary glands, oral mucosa, C. dental pain (e.g., attributed to irreversible pulpitis, or attributed to symptomatic apical periodontitis), C. neuropathic orofacial pain (e.g., TN or other cranial neuralgias), CH or COP attributed to chronic secondary temporomandibular disorders (e.g., chronic secondary orofacial muscle pain)
C. secondary visceral pain	(i) CVP from persistent inflammation	Each one could be in the head or neck region, in the thoracic region, in the abdominal region, in the pelvic region
	(ii) CVP from vascular mechanism	
	(iii) CVP from mechanical factors	
C. secondary musculoskeletal pain	(i) CMSP from persistent inflammation	(i) Due to infection, due to crystal deposition, due to autoimmune and autoinflammatory disorders
	(ii) CMSP associated with structural changes	(ii) CMSP associated with osteoarthritis, CMSP associated with spondylosis, CP after musculoskeletal injury
	(iii) CMSP associated with a disease of the nervous system	(iii) CMSP associated with Parkinson's diseases, CMSP associated with multiple sclerosis, CMSP associated with peripheral neurologic disease

C.: chronic, CRPS: chronic regional pain syndrome, CP: chronic pain, CCNP: chronic central neuropathic pain, CH: chronic headache, COP: chronic orofacial pain, and CVP: chronic visceral pain, CMSP: chronic musculoskeletal pain. Underlined: conditions included in this review.

**Table 3:** HBOT indications [9].

Indications for hyperbaric oxygen therapy

Air or gas embolisms
Carbon monoxide poisoning
Clostridial myositis and myonecrosis
Crush injury, compartment syndrome and other acute traumatic ischemia
Decompression sickness
Arterial insufficiencies (central retinal artery occlusion, enhancement of healing in selected problem wounds)
Severe anemia
Intracranial abscess
Necrotizing soft tissue infections
Refractory osteomyelitis
Delayed radiation injury (soft tissue and bony necrosis)
Acute thermal burn injury
Idiopathic sudden sensorineural hearing loss

**Table 4:** Side effects of hyperbaric oxygen therapy [10].

Middle ear barotrauma, sinus squeeze, claustrophobia, progressive myopia, pulmonary barotrauma, seizures	

**Table 5:** HBOT and neuropathic pain–animal studies.

First author, year	Article title	Pain model	Pain outcomes	HBO (depth, FiO2, duration, *n* of sessions)	Comparator	Timing of assessments, follow-up	Results	
							Pain outcomes	Other outcomes
Li, F et al., 2011 [17]	Hyperbaric oxygenation therapy alleviates chronic constrictive injury-induced neuropathic pain and reduces tumor necrosis factor-alpha production	Chronic constrictive injury of the sciatic nerve (CCI) in rats	(i) Mechanical allodynia (MWT) (ii) Cold allodynia	2.4 ATA 100% FiO_2_ 60 min 7 sessions (POD1-7)	1 ATA Room air 60 min 7 sessions (POD1-7)	MWT and cold allodynia tests on POD 4 and POD 7	Compared to the CCI-only group, HBOT-treated rats exhibited a significant increase in MWT and decrease in cold allodynia response frequency at day 4 and day 7	CCI-induced significant increase in TNF-*α* content in the sciatic nerve at days 4 and 7. This increase was significantly reduced in HBOT groups to near the level of sham rats

Gu, N et al., 2012 [18]	Hyperbaric oxygen therapy attenuates neuropathic hyperalgesia in rats and idiopathic trigeminal neuralgia in patients	Chronic constrictive injury of the sciatic nerve (CCI) in rats	(i) Mechanical allodynia (MWT) (ii) Thermal allodynia (TWL)	1.5, 2.0, and 3.0 ATA 100% FiO_2_ 70 min 7 sessions (POD1-7) 2 additional HBOT tx groups at 3.0 ATA receiving 3 sessions (POD14-16) and 7 sessions (POD 14–20)	1.0 ATA Room air 70 min 7 sessions	Multiple assessments of MWT and TWL from POD 0 to 28	Compared to the CCI-only, 1.5 ATA, and 2.0 ATA HBOT groups, the 3.0 ATA HBOT group demonstrated a significant increase in MWT and TWL. The effect persistent throughout the assessment period (POD28). When HBOT treatment (3.0 ATA) was delayed for 2 weeks following CCI (only 3 HBOT sessions); there was a significant but transient (∼1 week) increase in MWT and TWL compared to CCI-only group. When HBOT treatments were extended to 7 sessions; this antinociceptive effect was sustained throughout the assessment period	Repetitive HBOT treatments suppressed the CCI-induced induction of c-fos and the activation of astrocytes in the rat spinal cord as well as the CCI-induced increased phosphorylation of NR2B, ERK, CaMKII, and CREB in the spinal cord

Thompson et al., 2009 [19]	Hyperbaric oxygen treatment decreases pain in two nerve injury models	L5 nerve root ligation and chronic constrictive injury of the sciatic nerve (CCI) in rats	(i) Mechanical allodynia (MWT)	2.4 ATA 100% FiO_2_ 90 min 14 sessions (POD1-14)	1.0 ATA Room air 100 min 14 sessions (POD1-14)	MWT immediately following daily HBOT treatments (POD1-14) and then daily assessments for POD15-19	Both CCI and L5 ligation groups exposed to HBOT demonstrated increased MWT at nearly every time point after the start of treatment compared to control rats and the effect persisted throughout the post-HBOT 5-day assessment period. Of the two HBOT groups, the CCI group responded to treatment sooner than the L5 ligation group and the treatment effect was maintained longer	

Gibbons et al, 2013 [20]	Involvement of brain opioid receptors in the antiallodynic effect of hyperbaric oxygen in rats with sciatic nerve crush-induced neuropathic pain	Sciatic nerve crush injury	(i) Mechanical allodynia (MWT)	3.5 ATA 100% FiO_2_ 60 min 1 session (on POD7)	1.0 ATA Room air 60 min 1 session (on POD7)	MWT, every other day, up until POD30	HBOT group demonstrated significant increase in mechanical threshold (as measured by AUC for changes in MWT from POD7-30) compared to CCI-only (control) rats and approached the threshold of sham	Intraventricular administration of naltrexone (following nerve crush injury and prior to HBOT treatment) completely blocked the antinociceptive effect of HBOT

Zhao et al, 2015 [21]	Hyperbaric oxygen treatment at various stages following chronic constriction injury produces different antinociceptive effects via regulation of P2X4R expression and apoptosis	Chronic constrictive injury of the sciatic nerve (CCI)	(i) Mechanical allodynia (MWT) (ii) Thermal allodynia (TWL)	2.0 ATA 100% FiO_2_ 60 min 5 sessions 3 treatment groups starting at different time points (HOB1 : POD1-5, HBO6 POD6-10, and HBO11 : POD11-15)	1.0 ATA Room air 60 min 5 sessions 3 control groups starting at different time points (POD1-5, POD6-10, and POD11-15)	MWT and TWL assessed on postop days 1, 3, 5, 7, 10, 14, and 21	HBO1⟶significant increase in MWT and TWL following HBOT treatment and sustained for 21 days compared to CCI control HBO6 ⟶ significant, but transient (<1 wk) improvement in MWT and TWL compared to CCI control HBO11 ⟶ significant increase in MWT and TWL following HBOT treatment sustained for 10d compared to CCI control	HBOT early after injury (HBO1 group) inhibited the CCI-induced increase in expression of P2X4R (a ligand-gated ion channel activated by ATP and involved in the generation and maintenance of neuropathic pain). HBOT late after injury (HBO11) inhibited CCI-induced apoptosis via downregulation of caspase-3

Zhao et al, 2014 [22]	Hyperbaric oxygen treatment produces an antinociceptive response phase and inhibits astrocyte activation and inflammatory response in a rat model of neuropathic pain	Chronic constrictive injury of the sciatic nerve (CCI)	(i) Mechanical allodynia (MWT) (ii) Thermal allodynia (TWL)	2 groups: 2.0 ATA and 2.5 ATA 100% FiO_2_ 60 min 7 sessions (POD1-7)	1.0ATA Room air 100 min 7 sessions (POD1-7)	Multiple daily MWT and TWL assessments throughout the HBOT treatment period: T0 = immediately following HBOT T1 = 1 hr post-HBOT T2 = 2 hr post-HBOT T3 = before entering the HBOT chamber (following day)	T0 = decreased MWT and shortened TWL in both HBOT groups immediately after the first two HBOT sessions suggesting a transient allodynia with HBOT compared to CCI controls T1 = increased MWT and lengthened TWL in both HBOT groups compared to CCI control during all 7 treatment days suggesting a rapid shift from transient allodynia to antinociceptive response following HBOT session. T3 = increased MWT and lengthened TWL only after 5 days of HBOT compared to CCI controls. This suggests that the antinociceptive response is initially transient but may become prolonged with repetitive HBOT treatments	After 7 d (but not 4 d) of HBOT, there was a significant decrease in the CCI-induced upregulation of IL-1b and IL-10 in the spinal cord. HBOT treatment groups also demonstrated a reduction in CCI-induced increase in GFAP-immunoreactive astrocytes at day 7 in the spinal dorsal horn. The results may suggest that repetitive HBOT suppresses proinflammatory (IL-1b) cytokines, expresses anti-inflammatory cytokines (IL-10), and decreases astrocyte activation in the spinal cord

Han et al., 2013 [23]	Effects of hyperbaric oxygen on pain-related behaviors and nitric oxide synthase expression in a rat model of neuropathic pain	Chronic constrictive injury of the sciatic nerve (CCI)	(i) Mechanical allodynia (MWT) (ii) Thermal allodynia (TWL)	2.4 ATA FiO2 >90% 60 min 1 session either before or after CCI (POD-1 or POD + 1)	No control chamber treatment	MWT and TWL assessed on postop days 1, 2, 3, 7, 14, 21, and 28	HBOT before or after CCI resulted in a significant increase in MWT compared to CCI controls. HBOT before CCI resulted in a significant increase in TWL compared to no HBOT. HBOT after CCI resulted in only a 14 d transient increase in TWL compared to CCI control. These results suggest that the antinociceptive effects of HBOT are more substantial when HBOT is given prior to the injury, rather than after injury	HBOT groups demonstrated a reduction in nNOS- and iNOS-positive neurons in the spinal cord at 28d compared to control CCI rats

Ding et al., 2018 [24]	Early hyperbaric oxygen effects on neuropathic pain and nitric oxide synthase isoforms in CCI rats	Chronic constrictive injury of the sciatic nerve (CCI)	(i) Mechanical allodynia (MWT) (ii) Thermal allodynia (TWL)	2.5 ATA FiO2 >90% 60 min 5 sessions (POD1-5)	1.0 ATA Room air 60 min 5 sessions	Daily MWT and TWL assessments (POD1-14)	HBOT group had significant increase in both MWT and TWL compared to CCI control that was sustained throughout the assessment period	CCI-induced expression of iNOS and nNOS mRNA and protein in the ipsilateral spinal dorsal horn starting 3d after injury. HBOT treatment causes a significant reduction in the increased expression of these mRNAs and proteins

Fu et al., 2017 [25]	Hyperbaric oxygenation alleviates chronic constriction injury- (CCI-) induced neuropathic pain and inhibits GABAergic neuron apoptosis in the spinal cord	Chronic constrictive injury of the sciatic nerve (CCI)	(i) Mechanical allodynia (MWT)	2.4 ATA 98%FiO2 60 min 14 sessions (POD1-14)	1.0 ATA RA 60 min 14 sessions (POD1-14)	MWT assessed on POD #0, 8, and 14	HBOT treatment caused a significant increase in MWT compared to CCI control rats on POD8 and POD14	CCI-induced an increase in apoptotic positive neurons, apoptotic GABA-positive neurons, cleaved caspase 3 positive neurons, and cytochrome C positive neurons on POD 8 and 14. HBOT treatment mitigated the increase for these outcomes at both time points. This suggests the beneficial effect of HBOT in CCI-induced neuropathic pain may be due to its inhibitory role in CCI-induced GABAergic neuron apoptosis via suppressing the mitochondrial apoptotic pathways in the spinal cord

CCI = chronic constriction injury of the sciatic nerve; MWT = mechanical withdrawal threshold; TWL = thermal withdrawal latency; POD = postoperative day; AUC = area under the curve.

**Table 6:** HBOT and neuropathic pain–human studies characteristics.

First author, year	Article title	Pain model	Study design, *n* patients	Inclusion criteria	Intervention (HBOT pressure, duration, *n* of sessions)	Comparator/control
Kiralp, 2004 [33]	Effectiveness of hyperbaric oxygen therapy in the treatment of complex regional pain syndrome	CRPS	RCT, *n* = 71 (37 intervention, 34 placebo)	Clinical CRPS, type I or II	2.4 ATA, 90 min x 15	Placebo: 2.4 ATA breathing air, 90 min x 15, once daily

Peach, 1995 [35]	Hyperbaric oxygen and the reflex sympathetic dystrophy syndrome: a case report	CRPS	Observational case reports, *n* = 1	Clinical CRPS, type I	2.8 ATA, 46 min *x*1 + 2.0 ATA, 90 min x 1 + 2.4 ATA, 90 min x 1	None

Williams, 2009 [36]	Chronic regional pain syndrome after subtalar arthrodesis is not prevented by early hyperbaric oxygen	CRPS	Observational case reports, *n* = 1	Clinical CRPS type I, Norman Harden and Bruehl diagnostic criteria	2.2 ATA, 90 min x 19	None

Katznelson, 2016 [37]	Successful treatment of lower limb complex regional pain syndrome following three weeks of hyperbaric oxygen therapy	CRPS	Observational case reports, *n* = 1	Clinical CRPS, type I	2.4 ATA, 90 min x 15	None

Binkley, 2019 [38]	Successful treatment of long standing complex regional pain syndrome with hyperbaric oxygen therapy	CRPS	Observational case reports, *n* = 1	Clinical CRPS, type I	2.4 ATA, 90 min x 40. Second course 7 months later, 2.0 ATA, 90 min x 33.	None

van Ophoven, 2004 [39]	Hyperbaric oxygen for the treatment of interstitial cystitis: long-term results of a prospective pilot study	IC	Observational prospective case series, *n* = 6	Symptom criteria of the National Institute of Diabetes, Digestive and Kidney Diseases for IC	2.4 ATA, 90 min x 30	None

van Ophoven, 2006 [40]	Safety and efficacy of hyperbaric oxygen therapy for the treatment of interstitial cystitis: A randomized, sham controlled, double-blind trial	IC	RCT, double-blind, sham controlled, *n* = 21 (14 intervention, 7 placebo)	Diagnostic criteria of the National Institute of Diabetes and Digestive and Kidney Diseases for IC	2.4, 90 min x 30	Placebo: 1.3 ATA breathing air, 90 min x 30, once daily

Tanaka, 2011 [41]	Hyperbaric oxygen therapy for painful bladder syndrome/interstitial cystitis resistant to conventional treatments: long-term results of a case series in Japan	IC	Observational prospective case series, *n* = 11	Diagnostic criteria of the National Institute of Diabetes and Digestive and Kidney Diseases for IC	2.0 ATA, 60 min x 10 (8 pts) or x 20 (3 pts)	None

Wenzler, 2017 [42]	Treatment of ulcerative compared to nonulcerative interstitial cystitis with hyperbaric oxygen: a pilot study	IC	Observational prospective pilot case series, *n* = 9	Diagnostic criteria of the National Institute of Diabetes and Digestive and Kidney Diseases for IC	2.2 ATA, 90 min x 30	None

Pritchard, 2011 [43]	Double-blind randomized phase II study of hyperbaric oxygen in patients with radiation-induced brachial plexopathy	RIBP	RCT, double-blind, sham controlled, *n* = 34 (17 intervention, 17 placebo)	Confirmation of RIBP, freedom from cancer recurrence, fitness for HBOT	2.4 ATA, 90 min x 30	Placebo: 2.4 ATA breathing 41% oxygen, 90 min x 30

Videtic, 1999 [44]	Hyperbaric oxygen corrects sacral plexopathy due to osteoradionecrosis appearing 15 years after pelvic irradiation	Sacral plexopathy	Observational case reports, *n* = 1	Clinical diagnosis	2.5 ATA, 90 min x 30	None

Stowe, 2020 [45]	Hyperbaric oxygen therapy for radiation-induced brachial plexopathy, a case report and literature review	RIBP	Observational case reports, *n* = 1	Clinical and radiographic diagnosis	2.4 ATA, 120 min x 30	None

Peng, 2012 [46]	Effect of hyperbaric oxygen therapy on patients with herpes zoster	PHN	RCT, not blinded, *n* = 68 (36 intervention, 32 control)	Clinical diagnosis of acute herpes zoster	2.2 ATA, 80 min x 30, twice a day + medical therapy [antiviral (acyclovir), nerve nutritive (mecobalamin), pain relief (tramadol), and antidepressive (nortriptyline)]	Controls: only medical therapy

Gu, 2012 [18]	Hyperbaric oxygen therapy attenuates neuropathic hyperalgesia in rats and idiopathic trigeminal neuralgia in patients	TN	RCT, *n* = 42 (22 intervention, 20 placebo)	Clinical diagnosis of idiopathic TN	1.8 ATA, 70 min x 10	Placebo: 1.03 ATA breathing air, 70 min x 10

*n* = number; HBOT = hyperbaric oxygen therapy; CRPS = chronic regional pain syndrome; IC = interstitial cystitis; RIBP = radiation-induced brachial plexopathy; PHN = postherpetic neuralgia; TN = trigeminal neuralgia; RCT = randomized controlled trial.

**Table 7:** Effect of HBOT on neuropathic pain patient outcomes.

First author, year, pain model (*n* patients)	Outcomes	Timing of assessments and follow-up	Results		Remarks and safety
			Subjective clinical outcomes	Objective clinical outcomes	
Kiralp, 2004, [33]CRPS (*n* = 71)	Clinical (pain (VAS), range of motion (ROM), edema (wrist circumference))	Before and after HBOT, 45 days F/U	Intervention: lower pain (*p* < 0.001); placebo: no improvements	Intervention: increased ROM and decreased edema (*p* < 0.001). Placebo: no improvements	Placebo received therapeutic oxygen dose
Peach, 1995, [35] CRPS (*n* = 1)	Clinical (pain, cyanosis)	Before and after each HBOT session	Decreased pain	Decreased cyanosis	HBOT started for another indication (CO poisoning)

William, 2009, [36] CRPS (*n* = 1)	Clinical (wound healing, pain)	Before and after HBOT	Increased pain, allodynia, new neuropathic features	Skin color, edema	(i) HBOT started for another indication (wound healing) did not prevent CRPS
					(ii) HBOT was not subsequentially used as treatment

Katznelson, 2016, [37] CRPS (*n* = 1)	Clinical (pain intensity (VAS), edema, skin discoloration, ROM); questionnaires (pain interference with everyday functioning (brief pain inventory (BPI)), mood and anxiety (hospital anxiety and depression scale (HADS)))	Before and after HBOT, 1- and 6-month F/U	Decreased pain (VAS from 7 to 3.2); marked BPI decrease (30% for general activity), HADS improvement (depression from 9 to 6, anxiety from 6 to 4)	Improvement of discoloration, swelling, ROM; Tinel's sign disappeared; 6-minute walk test improvement (20%). Decreased medications to no medications required	

Binkley, 2019, [38] CRPS (*n* = 1)	Clinical (pain (VAS), edema, skin discoloration, ROM, stiffness, tremor; steroids side effects); medication doses; quality of life (QoL)	Before and after both treatments, 3- and 6-month F/U	Improvement of all clinical findings. Improved QoL	Decreased steroid dose	Mild claustrophobia
van Ophoven, 2004, [39] IC (*n* = 6)	Clinical (pain (VAS), symptoms severity (O'Leary-Sant ICSI), including urgency, nocturia, frequency); well-being (PGAF); satisfaction with HBOT; bladder biopsy	Before and after HBOT, F/U: every 3 months for 15 months after HBOT	4 responders: decreased pain (from 2–9.7 to 0.3–3), decreased symptom severity ICSI (from 23–35 to 5–16 after HBOT and 8–24 at 15-month F/U). Improved well-being (PGAF) and ICSI satisfied 2 nonresponders: no F/U. Nonsatisfied	(i) Improved ICSI (ii) Biopsy: nonulcerative (early) IC: 1 responder vs 1 nonresponder. Ulcerative (late) IC: 3 responders vs 1 nonresponder	(i) No support to the hypothesis that HBOT benefits more late-IC than early-IC (ii) 1 mild Eustachian disfunction
van Ophoven, 2006, [40] IC (*n* = 21)	Primary outcome: efficacy (global response assessment (GRA) questionnaire). Secondary outcomes: pain VAS, urgency (functional bladder capacity), frequency; symptoms severity (O'Leary-Sant ICSI); satisfaction	Before and after HBOT, 3- and 12-month F/U	Intervention: 3 responders (*p* < 0.52). At 12-month F/U, 3 patients (21.4%) reported treatment response. Decrease of baseline urgency intensity (from 60.2 +/- 15.0 to 49.9 +/- 35.2 mm, *p* < 0.05), decrease of pain (from 4.3 +/- 2 to 3.1 +/- 2, *p* < 0.05). Controls: no responders; no parameters improved compared to baseline	Intervention: ICSI decreased (from 25.7 to 19.9 points). Controls: no improvements	Conclusion: HBOT provides sustained decrease of IC symptoms with a discordant profile regarding the peak amelioration of symptoms compared to placebo

Tanaka, 2011, [41] IC (*n* = 11)	Efficacy (ICSI improvement > = 1), clinical (pain (VAS), urgency (VAS)); endoscopic findings	Before and after HBOT, 12-month F/U, variable F/U up to 50 months	7 responders, significant improvement in all measures (pain VAS from 7.7 ± 1.0 to 3.4 ± 2.5; urgency VAS from 6.6 ± 0.9 to 4.3 ± 2.4); sustained at F/U	(i) Improved ICSI (from 26.7 ± 7.0 to 18.7 ± 7.4 (*p* < 0.05)) (ii) Biopsy: 3 of the 4 nonresponders had nonulcerative IC	1 mild Eustachian disfunction, 3 mild exudative otitis media

Wenzler, 2017, [42] IC (*n* = 9)	Primary outcome: efficacy (GRA). Secondary: clinical symptoms (voiding diary, ICSI and ICPI questionnaires, VAS); cystoscopic appearance	Before HBOT, F/U after HBO: 2 weeks, months 3, 6, 12	5 responders, 1 nonresponder, 3 withdrew but considered nonresponder. Responders: GRA improved; VAS (1.5 points) improvement; voiding nonsignificant	(i) Improved ICSI, ICPI (1.5 points) (ii) Biopsy: 2 out of 3 ulcerative IC improved	(i) Nonulcerative IC (1 pt): marked improvement/resolution; ulcerative IC (4 pts): mild to moderate improvement (ii) No adverse events

Pritchard, 2001, [43] RIBP (*n* = 34)	Primary endpoint: warm sensory threshold. Secondary: heat pain threshold, cool sensation threshold, routine neurophysiological tests, pain (McGill pain questionnaire), and QoL (MOS SF-36 questionnaire)	Before HBOT, 1-week F/U, 12-month F/U	No significant difference between groups	No significant difference between groups. Intervention: nonsignificant improvement of warm sensory threshold	(i) Placebo protocol is HBOT (therapeutic itself), not a real placebo. (ii) Anecdotal evidence of improvement in longstanding arm lymphedema is an unexpected outcome of this study

Videtic, 1999, [44] sacral plexopathy (*n* = 1)	Clinical (pain), medication doses	Before and after HBOT, 12-month F/U	Marked pain improvement (from severe to none)	Marked reduction on medications (from high-dose multimodal to none)	HBOT started for another indication (ORN)

Stowe, 2020, [45] RIBP (*n* = 1)	Clinical (pain, ROM, neuroexam (sensory, motor)); imaging (brachial plexus MRI)	Before HBOT, F/U at 2, 6, and 13 months	Pain: from sever to none	(i) ROM: from decreased to full (ii) Imaging: significantly decreased abnormal enhancement	Benefit could be related to a longer HBOT (120 min) compared to other literature (90 min) and to an early diagnosis and treatment
Peng, 2012, [46] PHN (*n* = 68)	Primary outcome: therapeutic efficacy (objective measures (period of blister resolution, scar formation time, and percentage of patients developing PHN), subjective assessments (pain-NPRS, depression questionnaire-HAMD))	Before and after HBOT, 6-month F/U	Intervention: therapeutic efficacy (97.2%), significantly higher than control group (81.5%) (*p* < 0.05). Significant reduction in persistent PHN development (HBOT 11.1% vs control 31.3%). Pain and depression scores decreased significantly in both groups but were significantly lower in the intervention group	Intervention: significant reduction in scar formation time (HBOT 11.1 days ± 4 vs control 14 days ± 4.3)	(i) Study outcomes were measured when by natural history one would expect the infection to have resolved (5 weeks) (ii) Small differences in pain after treatment could be not clinically significant (ii) Although this is a positive study for HBOT in PHN, further studies are needed with chronic PHN (>3 months from onset) and longer follow-up periods

Gu, 2012, [18] TN (*n* = 42)	Primary outcome: changes in pain based on objective measure (changes in carbamazepine dose) and subjective assessments (pain VAS)	Before and after HBOT, 6-month F/U. 35 pts completed the study	Intervention group: VAS significantly decreased when comparing within-group to baseline and between-groups to the sham treatment, up to 6-month F/U	Intervention group: significant decrease in carbamazepine dose; significant lower dose for 60 days after HBOT than placebo; the lower dose was kept up to 90 days after HBOT	(i) A placebo effect was shown in the study (carbamazepine doses and VAS were decreased in the sham group, although the decrease was to a lesser degree than the treatment group) (ii) This study also implicates other cranial neuralgias as possible indications for HBOT

*n* = number; HBOT = hyperbaric oxygen therapy; CRPS = chronic regional pain syndrome; IC = interstitial cystitis; RIBP = radiation-induced brachial plexopathy; PHN = postherpetic neuralgia; TN = trigeminal neuralgia; VAS = visual analogue scale; ROM = range of motion; QoL = quality of life, ICSI = interstitial cystitis symptom index; ICPI = interstitial cystitis problem index; PGAF = patient global assessment form; MOS SF-36 = medical outcomes study, 36-Item Short Form Health Survey; MRI = magnetic resonance imaging; NPRS = numeric pain rating scale; HAMD= Hamilton depression rating scale; F/U = follow-up; ORN = osteoradio necrosis.

## Data Availability

Data are available in Supplementary Materials and are anyway available upon request to the corresponding author.

## Supplementary Materials

Supplementary Materials The supplementary materials include the Medline search strategy. .
