# Supplementary material for: Mechanistic Rationale and Clinical Efficacy of Hyperbaric Oxygen Therapy in Chronic Neuropathic Pain: An Evidence-Based Narrative Review
Source: Pain Res Manag. 2021 Apr 22;2021:8817504. doi: 10.1155/2021/8817504 (PMC8084668; doi:10.1155/2021/8817504)
Supplement: Supplementary Materials — The supplementary materials include the Medline search strategy. . [file 8817504.f1.docx]

**
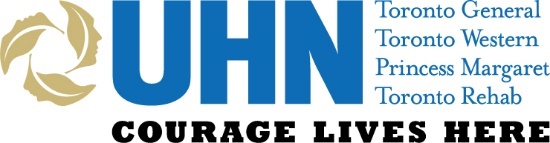
**

UHN Health Sciences Libraries

Literature Search Results

For: Drs Rita Katznelson, Simone Schiavo, and Julian DeBacker

Department: Anesthesia / Hyperbaric

Date Completed: March 21, 2020

Tel:

Fax:

Email:

**Attached is your search for**:

1. Scoping review searches for Hyperbaric Oxygen Therapy and Neuropathic Pain; limited to English language.

**The databases searched were**:

1. [Medline](#Medline); 2. [Medline In-Process/ePubs](#Medline_In_Process); 3. [Embase](#Embase); 4. [CCTR](#CCTR); 5. [CDSR](#CDSR); 6. [Web of Science](#Web_of_Science); 7. [Scopus](#Scopus); 8. [ClinicalTrials.Gov](#ClinicalTrials_Gov); 9. [WHO ICTRP](#WHO_ICTRP); 10. [ProQuest Dissertations & Theses Global](#ProQuest_Dissertations_Theses_Global)

**RESULTS & STRATEGY USED**: *see following*

**Search Completed By:** Marina Englesakis, Information Specialist

You may contact me either by telephone at (416) 340-4800 x3022 or via e-mail at [*marina.englesakis@uhn.ca*](mailto:marina.englesakis@uhn.ca)*.*

It is important that you are satisfied with your search results.

If you have any questions regarding this search, or if the results were not satisfactory, please do not hesitate in contacting me.

To request items not available in our library system, an INTERLIBRARY LOAN REQUEST FORM can be obtained from the library’s circulation desk or through the Virtual Library (<http://www.uhn.ca/Education/libraries/ill.asp> ). Any questions regarding our Document Delivery Service can be directed to Caleb by telephone at 416-340-4121 or by email at [Caleb.Nault@uhn.ca](mailto:Caleb.Nault@uhn.ca).

For any other circulation inquiries:

Toronto General Hospital Library: (416) 340-3429

Toronto Western Hospital Library: (416) 603-5750

Toronto Rehab Library: (416) 597-3422, ext. 3050

Princess Margaret Library: (416) 946-4482

Search strategy saved as 2020-03-21 RK SS HBOT and Neuropathic Pain - Searches

Contents

[Medline 2](#_Toc29726454)

[Medline In-Process 6](#_Toc29726455)

[Embase 9](#_Toc29726456)

[CCTR 13](#_Toc29726457)

[CDSR 17](#_Toc29726458)

[Web of Science 20](#_Toc29726459)

[Scopus 21](#_Toc29726460)

[ClinicalTrials.Gov 22](#_Toc29726461)

[WHO ICTRP 23](#_Toc29726462)

[ProQuest Dissertations & Theses Global 23](#_Toc29726463)

# Medline

Ovid MEDLINE(R) 1946 to March 21, 2020

| **#** | **Searches** | **Results** |
| --- | --- | --- |
| 1 | Hyperbaric Oxygenation/ [ MeSH ] | 11689 |
| 2 | "hyperbaric?? oxygen*".mp. | 13065 |
| 3 | high pressure chamber?.mp. | 62 |
| 4 | high pressure oxygen*.mp. | 199 |
| 5 | high tension o2.mp. | 0 |
| 6 | high tension oxygen*.mp. | 2 |
| 7 | hyperbaric environment?.mp. | 224 |
| 8 | hyperbaric room?.mp. | 6 |
| 9 | hyperbaric unit?.mp. | 37 |
| 10 | (hyperbaric? adj1 O2).mp. | 83 |
| 11 | (hyperbaric? adj1 oxygen*).mp. | 13068 |
| 12 | (hyperbaric? adj2 chamber?).mp. | 862 |
| 13 | (hyperbaric? adj2 medical*).mp,jw. | 95 |
| 14 | (hyperbaric? adj2 medicine).mp,jw. | 1890 |
| 15 | (hyperbaric? adj2 therap*).mp,jw. | 3473 |
| 16 | positive pressure chamber?.mp. | 1 |
| 17 | positive pressure room?.mp. | 3 |
| 18 | hbo.mp. | 2595 |
| 19 | hbo2.mp. | 1215 |
| 20 | hb-o2.mp. | 169 |
| 21 | hbot.mp. | 571 |
| 22 | or/1-21 [ Hyperbaric Oxygen Therapy & Related Terms ] | 15869 |
| 23 | exp Neuralgia/ | 19857 |
| 24 | exp Mononeuropathies/ | 19749 |
| 25 | exp Polyneuropathies/ | 27030 |
| 26 | exp Nerve Compression Syndromes/ | 21755 |
| 27 | exp Central Nervous System/ and exp "Wounds and Injuries"/ | 44271 |
| 28 | exp Central Nervous System/ and exp Pain/ | 23899 |
| 29 | exp Central Nervous System/in [Injuries] | 9210 |
| 30 | exp Complex Regional Pain Syndromes/ | 5469 |
| 31 | exp Peripheral Nerve Injuries/ | 6521 |
| 32 | exp Peripheral Nervous System Diseases/ | 144878 |
| 33 | exp Peripheral Nervous System/ and exp "Wounds and Injuries"/ | 25791 |
| 34 | exp Peripheral Nervous System/ and exp Pain/ | 23936 |
| 35 | exp Peripheral Nervous System/in [Injuries] | 13645 |
| 36 | Causalgia/ | 674 |
| 37 | Piriformis Muscle Syndrome/ | 126 |
| 38 | Reflex Sympathetic Dystrophy/ | 3562 |
| 39 | (central adj2 pain*).mp. | 2182 |
| 40 | (chemo* induced adj3 pain*).mp. | 248 |
| 41 | (chemo-induced adj3 pain*).mp. | 0 |
| 42 | (chemotherapy-induced adj3 pain*).mp. | 240 |
| 43 | (deafferentation adj2 pain*).mp,kw. | 297 |
| 44 | (dysa?sthetic adj2 pain*).mp,kw. | 7 |
| 45 | (entrap* adj3 syndrom*).mp,kw. | 986 |
| 46 | (maladapt* adj2 pain*).mp,kw. | 117 |
| 47 | (mal-adapt* adj2 pain*).mp,kw. | 2 |
| 48 | (mononeurit* adj1 multiple*).mp,kw. | 600 |
| 49 | (mono-neurit* adj1 multiple*).mp,kw. | 3 |
| 50 | (Morton* adj1 neuroma*).mp. | 315 |
| 51 | (nerve? adj12 pals???).mp,kw. | 13838 |
| 52 | (nerve? adj2 damag*).mp,kw. | 6834 |
| 53 | (nerve? adj2 injur*).mp,kw. | 30831 |
| 54 | (nerve? adj2 sensitiv*).mp,kw. | 1180 |
| 55 | (nerve? adj3 entrap*).mp,kw. | 2007 |
| 56 | (neural adj2 damag*).mp,kw. | 1603 |
| 57 | (neural adj2 injur*).mp,kw. | 1532 |
| 58 | (neural adj3 entrap*).mp,kw. | 37 |
| 59 | (neural adj3 sensitiv*).mp,kw. | 976 |
| 60 | (neuro* adj2 pain*).mp,kw. | 23162 |
| 61 | (neuro* adj2 sensitiv*).mp,kw. | 6847 |
| 62 | (peripheral adj2 nerve? adj2 injur*).mp. | 9897 |
| 63 | (peripheral* adj1 mononeurit*).mp,kw. | 7 |
| 64 | (peripheral* adj1 mono-neurit*).mp,kw. | 0 |
| 65 | (peripheral* adj1 neurit*).mp,kw. | 262 |
| 66 | (peripheral* adj1 polyneurit*).mp,kw. | 49 |
| 67 | (peripheral* adj1 poly-neurit*).mp,kw. | 0 |
| 68 | (phantom limb? adj2 pain*).mp. | 883 |
| 69 | (postamputat* adj2 pain*).mp. | 77 |
| 70 | (post-amputat* adj2 pain*).mp. | 38 |
| 71 | (postherpetic adj3 pain*).mp,kw. | 333 |
| 72 | (post-herpetic adj3 pain*).mp,kw. | 127 |
| 73 | (poststroke adj2 pain*).mp. | 171 |
| 74 | (post-stroke adj2 pain*).mp. | 238 |
| 75 | (pudendal adj2 entrap*).mp. | 51 |
| 76 | (radiation* induced adj3 pain*).mp. | 47 |
| 77 | (radiation-induced adj3 pain*).mp. | 47 |
| 78 | (radiotherapy-induced adj3 pain*).mp. | 3 |
| 79 | (somatosensory and pain*).mp,kw. | 5019 |
| 80 | (surgery-induced adj3 pain*).mp. | 17 |
| 81 | (surgically-induced adj3 pain*).mp. | 18 |
| 82 | (trauma* adj3 nerve? trunk?).mp. | 7 |
| 83 | allodynia*.mp,kw. | 7542 |
| 84 | allodynic*.mp,kw. | 650 |
| 85 | arthralgia*.mp. | 14084 |
| 86 | arthrodynia*.mp. | 9 |
| 87 | causalgi??.mp,kw. | 925 |
| 88 | cephalalgia?.mp. | 711 |
| 89 | cephalodynia*.mp. | 1 |
| 90 | cervicalgia*.mp. | 112 |
| 91 | cervicodynia*.mp. | 9 |
| 92 | cranialgia*.mp. | 0 |
| 93 | dorsalgia*.mp. | 97 |
| 94 | dysaesthesia*.mp,kw. | 355 |
| 95 | dysaesthesic*.mp,kw. | 0 |
| 96 | dysesthesia*.mp,kw. | 1761 |
| 97 | dysesthetic*.mp,kw. | 157 |
| 98 | Herpes Zoster*.mp,kw. | 13571 |
| 99 | hyper?esthesi*.mp,kw. | 1507 |
| 100 | hyperalgesia*.mp,kw. | 16410 |
| 101 | hyperpathia*.mp,kw. | 153 |
| 102 | hypo?esthesi*.mp,kw. | 1317 |
| 103 | mononeuropath???.mp,kw. | 1720 |
| 104 | mono-neuropath???.mp,kw. | 13 |
| 105 | multiple sclerosis.mp. | 71246 |
| 106 | myodynia*.mp. | 5 |
| 107 | nerve root disorder?.mp. | 8 |
| 108 | neuralgi*.mp,kw. | 24840 |
| 109 | neuritis.mp. | 15914 |
| 110 | neuropathic.mp,kw. | 23518 |
| 111 | neuropathies.mp,kw. | 28996 |
| 112 | neuropathy.mp,kw. | 63897 |
| 113 | nonnociceptive.mp,kw. | 129 |
| 114 | non-nociceptive.mp,kw. | 542 |
| 115 | numbness.mp,kw. | 7148 |
| 116 | paraesthesia*.mp,kw. | 1775 |
| 117 | paresthesia*.mp,kw. | 10814 |
| 118 | perineuritis.mp. | 123 |
| 119 | piriformis muscle syndrome?.mp,kw. | 148 |
| 120 | polyarthralgia*.mp. | 763 |
| 121 | polymyalgia*.mp. | 3043 |
| 122 | polyneuropath???.mp,kw. | 15446 |
| 123 | poly-neuropath???.mp,kw. | 15 |
| 124 | polyradiculitis.mp. | 528 |
| 125 | polyradiculoneuropath???.mp. | 5750 |
| 126 | polyradiculopathies.mp. | 19 |
| 127 | polyradiculopathy.mp. | 2775 |
| 128 | postherpetic.mp,kw. | 2226 |
| 129 | post-herpetic.mp,kw. | 780 |
| 130 | radicular pain*.mp. | 2047 |
| 131 | radiculitis.mp. | 751 |
| 132 | radiculopathies.mp. | 385 |
| 133 | radiculopathy.mp. | 7745 |
| 134 | sciatica.mp. | 6187 |
| 135 | shingles.mp,kw. | 1134 |
| 136 | trigeminal neuralgi*.mp. | 7533 |
| 137 | or/23-136 [ Neuropathic Pain & Related Terms - December 31 2019 ] | 459790 |
| 138 | 22 and 137 [ HBOT + Neuropathic Pain ] | 657 |
| 139 | limit 138 to english language | 546 |
| 140 | remove duplicates from 139 | 542 |

# Medline In-Process

Ovid MEDLINE(R) Epub Ahead of Print and In-Process & Other Non-Indexed Citations March 21, 2020

| **#** | **Searches** | **Results** |
| --- | --- | --- |
| 1 | Hyperbaric Oxygenation/ [ MeSH ] | 0 |
| 2 | "hyperbaric?? oxygen*".mp. | 847 |
| 3 | high pressure chamber?.mp. | 15 |
| 4 | high pressure oxygen*.mp. | 24 |
| 5 | high tension o2.mp. | 0 |
| 6 | high tension oxygen*.mp. | 1 |
| 7 | hyperbaric environment?.mp. | 21 |
| 8 | hyperbaric room?.mp. | 1 |
| 9 | hyperbaric unit?.mp. | 7 |
| 10 | (hyperbaric? adj1 O2).mp. | 2 |
| 11 | (hyperbaric? adj1 oxygen*).mp. | 848 |
| 12 | (hyperbaric? adj2 chamber?).mp. | 54 |
| 13 | (hyperbaric? adj2 medical*).mp,jw. | 11 |
| 14 | (hyperbaric? adj2 medicine).mp,jw. | 160 |
| 15 | (hyperbaric? adj2 therap*).mp,jw. | 548 |
| 16 | positive pressure chamber?.mp. | 0 |
| 17 | positive pressure room?.mp. | 1 |
| 18 | hbo.mp. | 301 |
| 19 | hbo2.mp. | 112 |
| 20 | hb-o2.mp. | 11 |
| 21 | hbot.mp. | 166 |
| 22 | or/1-21 [ Hyperbaric Oxygen Therapy & Related Terms ] | 1255 |
| 23 | exp Neuralgia/ | 0 |
| 24 | exp Mononeuropathies/ | 0 |
| 25 | exp Polyneuropathies/ | 0 |
| 26 | exp Nerve Compression Syndromes/ | 0 |
| 27 | exp Central Nervous System/ and exp "Wounds and Injuries"/ | 0 |
| 28 | exp Central Nervous System/ and exp Pain/ | 0 |
| 29 | exp Central Nervous System/in [Injuries] | 0 |
| 30 | exp Complex Regional Pain Syndromes/ | 0 |
| 31 | exp Peripheral Nerve Injuries/ | 0 |
| 32 | exp Peripheral Nervous System Diseases/ | 0 |
| 33 | exp Peripheral Nervous System/ and exp "Wounds and Injuries"/ | 0 |
| 34 | exp Peripheral Nervous System/ and exp Pain/ | 0 |
| 35 | exp Peripheral Nervous System/in [Injuries] | 0 |
| 36 | Causalgia/ | 0 |
| 37 | Piriformis Muscle Syndrome/ | 0 |
| 38 | Reflex Sympathetic Dystrophy/ | 0 |
| 39 | (central adj2 pain*).mp. | 362 |
| 40 | (chemo* induced adj3 pain*).mp. | 73 |
| 41 | (chemo-induced adj3 pain*).mp. | 0 |
| 42 | (chemotherapy-induced adj3 pain*).mp. | 72 |
| 43 | (deafferentation adj2 pain*).mp,kw. | 19 |
| 44 | (dysa?sthetic adj2 pain*).mp,kw. | 0 |
| 45 | (entrap* adj3 syndrom*).mp,kw. | 151 |
| 46 | (maladapt* adj2 pain*).mp,kw. | 21 |
| 47 | (mal-adapt* adj2 pain*).mp,kw. | 0 |
| 48 | (mononeurit* adj1 multiple*).mp,kw. | 69 |
| 49 | (mono-neurit* adj1 multiple*).mp,kw. | 2 |
| 50 | (Morton* adj1 neuroma*).mp. | 43 |
| 51 | (nerve? adj12 pals???).mp,kw. | 2266 |
| 52 | (nerve? adj2 damag*).mp,kw. | 966 |
| 53 | (nerve? adj2 injur*).mp,kw. | 3589 |
| 54 | (nerve? adj2 sensitiv*).mp,kw. | 66 |
| 55 | (nerve? adj3 entrap*).mp,kw. | 374 |
| 56 | (neural adj2 damag*).mp,kw. | 218 |
| 57 | (neural adj2 injur*).mp,kw. | 262 |
| 58 | (neural adj3 entrap*).mp,kw. | 8 |
| 59 | (neural adj3 sensitiv*).mp,kw. | 151 |
| 60 | (neuro* adj2 pain*).mp,kw. | 4565 |
| 61 | (neuro* adj2 sensitiv*).mp,kw. | 506 |
| 62 | (peripheral adj2 nerve? adj2 injur*).mp. | 1031 |
| 63 | (peripheral* adj1 mononeurit*).mp,kw. | 3 |
| 64 | (peripheral* adj1 mono-neurit*).mp,kw. | 0 |
| 65 | (peripheral* adj1 neurit*).mp,kw. | 79 |
| 66 | (peripheral* adj1 polyneurit*).mp,kw. | 0 |
| 67 | (peripheral* adj1 poly-neurit*).mp,kw. | 0 |
| 68 | (phantom limb? adj2 pain*).mp. | 158 |
| 69 | (postamputat* adj2 pain*).mp. | 15 |
| 70 | (post-amputat* adj2 pain*).mp. | 16 |
| 71 | (postherpetic adj3 pain*).mp,kw. | 46 |
| 72 | (post-herpetic adj3 pain*).mp,kw. | 21 |
| 73 | (poststroke adj2 pain*).mp. | 27 |
| 74 | (post-stroke adj2 pain*).mp. | 44 |
| 75 | (pudendal adj2 entrap*).mp. | 11 |
| 76 | (radiation* induced adj3 pain*).mp. | 9 |
| 77 | (radiation-induced adj3 pain*).mp. | 9 |
| 78 | (radiotherapy-induced adj3 pain*).mp. | 1 |
| 79 | (somatosensory and pain*).mp,kw. | 484 |
| 80 | (surgery-induced adj3 pain*).mp. | 1 |
| 81 | (surgically-induced adj3 pain*).mp. | 5 |
| 82 | (trauma* adj3 nerve? trunk?).mp. | 0 |
| 83 | allodynia*.mp,kw. | 1096 |
| 84 | allodynic*.mp,kw. | 77 |
| 85 | arthralgia*.mp. | 935 |
| 86 | arthrodynia*.mp. | 0 |
| 87 | causalgi??.mp,kw. | 46 |
| 88 | cephalalgia?.mp. | 89 |
| 89 | cephalodynia*.mp. | 0 |
| 90 | cervicalgia*.mp. | 31 |
| 91 | cervicodynia*.mp. | 1 |
| 92 | cranialgia*.mp. | 0 |
| 93 | dorsalgia*.mp. | 10 |
| 94 | dysaesthesia*.mp,kw. | 19 |
| 95 | dysaesthesic*.mp,kw. | 0 |
| 96 | dysesthesia*.mp,kw. | 248 |
| 97 | dysesthetic*.mp,kw. | 12 |
| 98 | Herpes Zoster*.mp,kw. | 1074 |
| 99 | hyper?esthesi*.mp,kw. | 83 |
| 100 | hyperalgesia*.mp,kw. | 1368 |
| 101 | hyperpathia*.mp,kw. | 8 |
| 102 | hypo?esthesi*.mp,kw. | 289 |
| 103 | mononeuropath???.mp,kw. | 156 |
| 104 | mono-neuropath???.mp,kw. | 6 |
| 105 | multiple sclerosis.mp. | 9347 |
| 106 | myodynia*.mp. | 0 |
| 107 | nerve root disorder?.mp. | 2 |
| 108 | neuralgi*.mp,kw. | 1887 |
| 109 | neuritis.mp. | 1269 |
| 110 | neuropathic.mp,kw. | 4203 |
| 111 | neuropathies.mp,kw. | 1269 |
| 112 | neuropathy.mp,kw. | 8079 |
| 113 | nonnociceptive.mp,kw. | 6 |
| 114 | non-nociceptive.mp,kw. | 29 |
| 115 | numbness.mp,kw. | 1581 |
| 116 | paraesthesia*.mp,kw. | 234 |
| 117 | paresthesia*.mp,kw. | 1167 |
| 118 | perineuritis.mp. | 42 |
| 119 | piriformis muscle syndrome?.mp,kw. | 10 |
| 120 | polyarthralgia*.mp. | 117 |
| 121 | polymyalgia*.mp. | 239 |
| 122 | polyneuropath???.mp,kw. | 1374 |
| 123 | poly-neuropath???.mp,kw. | 9 |
| 124 | polyradiculitis.mp. | 6 |
| 125 | polyradiculoneuropath???.mp. | 169 |
| 126 | polyradiculopathies.mp. | 5 |
| 127 | polyradiculopathy.mp. | 47 |
| 128 | postherpetic.mp,kw. | 292 |
| 129 | post-herpetic.mp,kw. | 156 |
| 130 | radicular pain*.mp. | 430 |
| 131 | radiculitis.mp. | 63 |
| 132 | radiculopathies.mp. | 43 |
| 133 | radiculopathy.mp. | 1160 |
| 134 | sciatica.mp. | 507 |
| 135 | shingles.mp,kw. | 180 |
| 136 | trigeminal neuralgi*.mp. | 784 |
| 137 | or/23-136 [ Neuropathic Pain & Related Terms - December 31 2019 ] | 39767 |
| 138 | 22 and 137 [ HBOT + Neuropathic Pain ] | 48 |
| 139 | limit 138 to english language | 48 |
| 140 | remove duplicates from 139 | 48 |

# Embase

Embase Classic+Embase 1947 to 2020 March 21

| **#** | **Searches** | **Results** |
| --- | --- | --- |
| 1 | hyperbaric oxygen therapy/ | 2466 |
| 2 | hyperbaric chamber/ | 444 |
| 3 | Hyperbaric Oxygenation/ [ MeSH ] | 2443 |
| 4 | "hyperbaric?? oxygen*".mp. | 20711 |
| 5 | high pressure chamber?.mp. | 108 |
| 6 | high pressure oxygen*.mp. | 376 |
| 7 | high tension o2.mp. | 0 |
| 8 | high tension oxygen*.mp. | 5 |
| 9 | hyperbaric environment?.mp. | 329 |
| 10 | hyperbaric room?.mp. | 10 |
| 11 | hyperbaric unit?.mp. | 67 |
| 12 | (hyperbaric? adj1 O2).mp. | 174 |
| 13 | (hyperbaric? adj1 oxygen*).mp. | 20720 |
| 14 | (hyperbaric? adj2 chamber?).mp. | 1563 |
| 15 | (hyperbaric? adj2 medical*).mp,jw. | 1328 |
| 16 | (hyperbaric? adj2 medicine).mp,jw. | 2413 |
| 17 | (hyperbaric? adj2 therap*).mp,jw. | 6974 |
| 18 | positive pressure chamber?.mp. | 3 |
| 19 | positive pressure room?.mp. | 14 |
| 20 | hbo.mp. | 3474 |
| 21 | hbo2.mp. | 1883 |
| 22 | hb-o2.mp. | 266 |
| 23 | hbot.mp. | 1042 |
| 24 | or/1-23 [ Hyperbaric Oxygen Therapy & Related Terms ] | 25002 |
| 25 | exp Central Nervous System/ and exp *"Wounds and Injuries"/ | 89565 |
| 26 | exp Central Nervous System/ and exp *Pain/ | 45845 |
| 27 | exp Complex Regional Pain Syndromes/ | 9933 |
| 28 | exp complex regional pain syndrome/ [Embase] | 9933 |
| 29 | exp Cumulative Trauma Disorder/ [Embase] | 21463 |
| 30 | exp Mononeuropathies/ | 78097 |
| 31 | exp Mononeuropathy/ [Embase] | 78097 |
| 32 | exp Nerve Compression Syndromes/ | 13836 |
| 33 | exp Nerve Compression/ [Embase] | 13836 |
| 34 | exp Neuralgia/ [MeSH & Embase] | 111002 |
| 35 | exp Neuropathic Pain/ [Embase] | 31564 |
| 36 | exp Peripheral Nervous System/ and exp *"Wounds and Injuries"/ | 34021 |
| 37 | exp Peripheral Nervous System/ and exp *Pain/ | 35072 |
| 38 | exp Peripheral Neuropathy/ [Embase] | 72849 |
| 39 | exp Polyneuropathies/ | 41586 |
| 40 | exp Polyneuropathy/ [Embase] | 41586 |
| 41 | Causalgia/ | 1246 |
| 42 | Herpes Zoster/ [Embase] | 24302 |
| 43 | Piriformis Muscle Syndrome/ | 111 |
| 44 | Piriformis Syndrome/ [Embase] | 230 |
| 45 | Reflex Sympathetic Dystrophy/ | 2650 |
| 46 | (central adj2 pain*).mp. | 3975 |
| 47 | (chemo* induced adj3 pain*).mp. | 458 |
| 48 | (chemo-induced adj3 pain*).mp. | 4 |
| 49 | (chemotherapy-induced adj3 pain*).mp. | 443 |
| 50 | (deafferentation adj2 pain*).mp,kw. | 461 |
| 51 | (dysa?sthetic adj2 pain*).mp,kw. | 21 |
| 52 | (entrap* adj3 syndrom*).mp,kw. | 1724 |
| 53 | (maladapt* adj2 pain*).mp,kw. | 185 |
| 54 | (mal-adapt* adj2 pain*).mp,kw. | 3 |
| 55 | (mononeurit* adj1 multiple*).mp,kw. | 1077 |
| 56 | (mono-neurit* adj1 multiple*).mp,kw. | 10 |
| 57 | (Morton* adj1 neuroma*).mp. | 563 |
| 58 | (nerve? adj12 pals???).mp,kw. | 25876 |
| 59 | (nerve? adj2 damag*).mp,kw. | 11387 |
| 60 | (nerve? adj2 injur*).mp,kw. | 61224 |
| 61 | (nerve? adj2 sensitiv*).mp,kw. | 1693 |
| 62 | (nerve? adj3 entrap*).mp,kw. | 3430 |
| 63 | (neural adj2 damag*).mp,kw. | 2438 |
| 64 | (neural adj2 injur*).mp,kw. | 2436 |
| 65 | (neural adj3 entrap*).mp,kw. | 61 |
| 66 | (neural adj3 sensitiv*).mp,kw. | 1314 |
| 67 | (neuro* adj2 pain*).mp,kw. | 50127 |
| 68 | (neuro* adj2 sensitiv*).mp,kw. | 9124 |
| 69 | (peripheral adj2 nerve? adj2 injur*).mp. | 12609 |
| 70 | (peripheral* adj1 mononeurit*).mp,kw. | 5 |
| 71 | (peripheral* adj1 mono-neurit*).mp,kw. | 0 |
| 72 | (peripheral* adj1 neurit*).mp,kw. | 607 |
| 73 | (peripheral* adj1 polyneurit*).mp,kw. | 30 |
| 74 | (peripheral* adj1 poly-neurit*).mp,kw. | 0 |
| 75 | (phantom limb? adj2 pain*).mp. | 1566 |
| 76 | (postamputat* adj2 pain*).mp. | 122 |
| 77 | (post-amputat* adj2 pain*).mp. | 104 |
| 78 | (postherpetic adj3 pain*).mp,kw. | 550 |
| 79 | (post-herpetic adj3 pain*).mp,kw. | 296 |
| 80 | (poststroke adj2 pain*).mp. | 287 |
| 81 | (post-stroke adj2 pain*).mp. | 559 |
| 82 | (pudendal adj2 entrap*).mp. | 107 |
| 83 | (radiation* induced adj3 pain*).mp. | 91 |
| 84 | (radiation-induced adj3 pain*).mp. | 91 |
| 85 | (radiotherapy-induced adj3 pain*).mp. | 16 |
| 86 | (somatosensory and pain*).mp,kw. | 7975 |
| 87 | (surgery-induced adj3 pain*).mp. | 25 |
| 88 | (surgically-induced adj3 pain*).mp. | 31 |
| 89 | (trauma* adj3 nerve? trunk?).mp. | 9 |
| 90 | allodynia*.mp,kw. | 16033 |
| 91 | allodynic*.mp,kw. | 939 |
| 92 | arthralgia*.mp. | 64050 |
| 93 | arthrodynia*.mp. | 17 |
| 94 | causalgi??.mp,kw. | 1608 |
| 95 | cephalalgia?.mp. | 1816 |
| 96 | cephalodynia*.mp. | 2 |
| 97 | cervicalgia*.mp. | 292 |
| 98 | cervicodynia*.mp. | 23 |
| 99 | cranialgia*.mp. | 4 |
| 100 | dorsalgia*.mp. | 187 |
| 101 | dysaesthesia*.mp,kw. | 616 |
| 102 | dysaesthesic*.mp,kw. | 1 |
| 103 | dysesthesia*.mp,kw. | 6179 |
| 104 | dysesthetic*.mp,kw. | 240 |
| 105 | Herpes Zoster*.mp,kw. | 29361 |
| 106 | hyper?esthesi*.mp,kw. | 2722 |
| 107 | hyperalgesia*.mp,kw. | 24020 |
| 108 | hyperpathia*.mp,kw. | 269 |
| 109 | hypo?esthesi*.mp,kw. | 2839 |
| 110 | mononeuropath???.mp,kw. | 4228 |
| 111 | mono-neuropath???.mp,kw. | 46 |
| 112 | multiple sclerosis.mp. | 142583 |
| 113 | myodynia*.mp. | 6 |
| 114 | nerve root disorder?.mp. | 19 |
| 115 | neuralgi*.mp,kw. | 34524 |
| 116 | neuritis.mp. | 27797 |
| 117 | neuropathic.mp,kw. | 53326 |
| 118 | neuropathies.mp,kw. | 17113 |
| 119 | neuropathy.mp,kw. | 201863 |
| 120 | nonnociceptive.mp,kw. | 172 |
| 121 | non-nociceptive.mp,kw. | 744 |
| 122 | numbness.mp,kw. | 15169 |
| 123 | paraesthesia*.mp,kw. | 4381 |
| 124 | paresthesia*.mp,kw. | 54131 |
| 125 | perineuritis.mp. | 250 |
| 126 | piriformis muscle syndrome?.mp,kw. | 86 |
| 127 | polyarthralgia*.mp. | 1435 |
| 128 | polymyalgia*.mp. | 6242 |
| 129 | polyneuropath???.mp,kw. | 31774 |
| 130 | poly-neuropath???.mp,kw. | 88 |
| 131 | polyradiculitis.mp. | 751 |
| 132 | polyradiculoneuropath???.mp. | 4340 |
| 133 | polyradiculopathies.mp. | 37 |
| 134 | polyradiculopathy.mp. | 607 |
| 135 | postherpetic.mp,kw. | 6428 |
| 136 | post-herpetic.mp,kw. | 1692 |
| 137 | radicular pain*.mp. | 5483 |
| 138 | radiculitis.mp. | 1840 |
| 139 | radiculopathies.mp. | 686 |
| 140 | radiculopathy.mp. | 14008 |
| 141 | sciatica.mp. | 7164 |
| 142 | shingles.mp,kw. | 2077 |
| 143 | trigeminal neuralgi*.mp. | 8585 |
| 144 | or/25-143 [ Neuropathic Pain & Related Terms - EMTree & MeSH; December 31 2019 ] | 903554 |
| 145 | 24 and 144 | 1712 |
| 146 | limit 145 to english language | 1497 |
| 147 | remove duplicates from 146 | 1481 |

# CCTR

Cochrane Central Register of Controlled Trials 2014 to Present

| **#** | **Searches** | **Results** |
| --- | --- | --- |
| 1 | hyperbaric oxygen therapy/ | 361 |
| 2 | hyperbaric chamber/ | 0 |
| 3 | Hyperbaric Oxygenation/ [ MeSH ] | 361 |
| 4 | "hyperbaric?? oxygen*".mp. | 1206 |
| 5 | high pressure chamber?.mp. | 1 |
| 6 | high pressure oxygen*.mp. | 19 |
| 7 | high tension o2.mp. | 0 |
| 8 | high tension oxygen*.mp. | 0 |
| 9 | hyperbaric environment?.mp. | 15 |
| 10 | hyperbaric room?.mp. | 1 |
| 11 | hyperbaric unit?.mp. | 1 |
| 12 | (hyperbaric? adj1 O2).mp. | 8 |
| 13 | (hyperbaric? adj1 oxygen*).mp. | 1207 |
| 14 | (hyperbaric? adj2 chamber?).mp. | 130 |
| 15 | (hyperbaric? adj2 medical*).mp,jw. | 14 |
| 16 | (hyperbaric? adj2 medicine).mp,jw. | 151 |
| 17 | (hyperbaric? adj2 therap*).mp,jw. | 691 |
| 18 | positive pressure chamber?.mp. | 0 |
| 19 | positive pressure room?.mp. | 0 |
| 20 | hbo.mp. | 265 |
| 21 | hbo2.mp. | 114 |
| 22 | hb-o2.mp. | 8 |
| 23 | hbot.mp. | 166 |
| 24 | or/1-23 [ Hyperbaric Oxygen Therapy & Related Terms ] | 1366 |
| 25 | exp Central Nervous System/ and exp *"Wounds and Injuries"/ | 237 |
| 26 | exp Central Nervous System/ and exp *Pain/ | 428 |
| 27 | exp Complex Regional Pain Syndromes/ | 274 |
| 28 | exp complex regional pain syndrome/ [Embase] | 0 |
| 29 | exp Cumulative Trauma Disorder/ [Embase] | 779 |
| 30 | exp Mononeuropathies/ | 979 |
| 31 | exp Mononeuropathy/ [Embase] | 979 |
| 32 | exp Nerve Compression Syndromes/ | 762 |
| 33 | exp Nerve Compression/ [Embase] | 0 |
| 34 | exp Neuralgia/ [MeSH & Embase] | 1427 |
| 35 | exp Neuropathic Pain/ [Embase] | 1427 |
| 36 | exp Peripheral Nervous System/ and exp *"Wounds and Injuries"/ | 296 |
| 37 | exp Peripheral Nervous System/ and exp *Pain/ | 1002 |
| 38 | exp Peripheral Neuropathy/ [Embase] | 4842 |
| 39 | exp Polyneuropathies/ | 443 |
| 40 | exp Polyneuropathy/ [Embase] | 443 |
| 41 | Causalgia/ | 18 |
| 42 | Herpes Zoster/ [Embase] | 463 |
| 43 | Piriformis Muscle Syndrome/ | 6 |
| 44 | Piriformis Syndrome/ [Embase] | 6 |
| 45 | Reflex Sympathetic Dystrophy/ | 201 |
| 46 | (central adj2 pain*).mp. | 669 |
| 47 | (chemo* induced adj3 pain*).mp. | 80 |
| 48 | (chemo-induced adj3 pain*).mp. | 0 |
| 49 | (chemotherapy-induced adj3 pain*).mp. | 74 |
| 50 | (deafferentation adj2 pain*).mp,kw. | 24 |
| 51 | (dysa?sthetic adj2 pain*).mp,kw. | 2 |
| 52 | (entrap* adj3 syndrom*).mp,kw. | 61 |
| 53 | (maladapt* adj2 pain*).mp,kw. | 40 |
| 54 | (mal-adapt* adj2 pain*).mp,kw. | 0 |
| 55 | (mononeurit* adj1 multiple*).mp,kw. | 8 |
| 56 | (mono-neurit* adj1 multiple*).mp,kw. | 0 |
| 57 | (Morton* adj1 neuroma*).mp. | 47 |
| 58 | (nerve? adj12 pals???).mp,kw. | 603 |
| 59 | (nerve? adj2 damag*).mp,kw. | 654 |
| 60 | (nerve? adj2 injur*).mp,kw. | 1567 |
| 61 | (nerve? adj2 sensitiv*).mp,kw. | 101 |
| 62 | (nerve? adj3 entrap*).mp,kw. | 122 |
| 63 | (neural adj2 damag*).mp,kw. | 67 |
| 64 | (neural adj2 injur*).mp,kw. | 56 |
| 65 | (neural adj3 entrap*).mp,kw. | 1 |
| 66 | (neural adj3 sensitiv*).mp,kw. | 71 |
| 67 | (neuro* adj2 pain*).mp,kw. | 5662 |
| 68 | (neuro* adj2 sensitiv*).mp,kw. | 486 |
| 69 | (peripheral adj2 nerve? adj2 injur*).mp. | 283 |
| 70 | (peripheral* adj1 mononeurit*).mp,kw. | 0 |
| 71 | (peripheral* adj1 mono-neurit*).mp,kw. | 0 |
| 72 | (peripheral* adj1 neurit*).mp,kw. | 57 |
| 73 | (peripheral* adj1 polyneurit*).mp,kw. | 0 |
| 74 | (peripheral* adj1 poly-neurit*).mp,kw. | 0 |
| 75 | (phantom limb? adj2 pain*).mp. | 209 |
| 76 | (postamputat* adj2 pain*).mp. | 19 |
| 77 | (post-amputat* adj2 pain*).mp. | 21 |
| 78 | (postherpetic adj3 pain*).mp,kw. | 260 |
| 79 | (post-herpetic adj3 pain*).mp,kw. | 97 |
| 80 | (poststroke adj2 pain*).mp. | 40 |
| 81 | (post-stroke adj2 pain*).mp. | 129 |
| 82 | (pudendal adj2 entrap*).mp. | 10 |
| 83 | (radiation* induced adj3 pain*).mp. | 31 |
| 84 | (radiation-induced adj3 pain*).mp. | 31 |
| 85 | (radiotherapy-induced adj3 pain*).mp. | 9 |
| 86 | (somatosensory and pain*).mp,kw. | 583 |
| 87 | (surgery-induced adj3 pain*).mp. | 8 |
| 88 | (surgically-induced adj3 pain*).mp. | 2 |
| 89 | (trauma* adj3 nerve? trunk?).mp. | 4 |
| 90 | allodynia*.mp,kw. | 692 |
| 91 | allodynic*.mp,kw. | 41 |
| 92 | arthralgia*.mp. | 5290 |
| 93 | arthrodynia*.mp. | 2 |
| 94 | causalgi??.mp,kw. | 41 |
| 95 | cephalalgia?.mp. | 49 |
| 96 | cephalodynia*.mp. | 0 |
| 97 | cervicalgia*.mp. | 43 |
| 98 | cervicodynia*.mp. | 2 |
| 99 | cranialgia*.mp. | 0 |
| 100 | dorsalgia*.mp. | 98 |
| 101 | dysaesthesia*.mp,kw. | 26 |
| 102 | dysaesthesic*.mp,kw. | 1 |
| 103 | dysesthesia*.mp,kw. | 267 |
| 104 | dysesthetic*.mp,kw. | 11 |
| 105 | Herpes Zoster*.mp,kw. | 1990 |
| 106 | hyper?esthesi*.mp,kw. | 120 |
| 107 | hyperalgesia*.mp,kw. | 1547 |
| 108 | hyperpathia*.mp,kw. | 6 |
| 109 | hypo?esthesi*.mp,kw. | 207 |
| 110 | mononeuropath???.mp,kw. | 67 |
| 111 | mono-neuropath???.mp,kw. | 2 |
| 112 | multiple sclerosis.mp. | 9730 |
| 113 | myodynia*.mp. | 2 |
| 114 | nerve root disorder?.mp. | 3 |
| 115 | neuralgi*.mp,kw. | 2718 |
| 116 | neuritis.mp. | 786 |
| 117 | neuropathic.mp,kw. | 4042 |
| 118 | neuropathies.mp,kw. | 1468 |
| 119 | neuropathy.mp,kw. | 10006 |
| 120 | nonnociceptive.mp,kw. | 6 |
| 121 | non-nociceptive.mp,kw. | 31 |
| 122 | numbness.mp,kw. | 1366 |
| 123 | paraesthesia*.mp,kw. | 401 |
| 124 | paresthesia*.mp,kw. | 3211 |
| 125 | perineuritis.mp. | 1 |
| 126 | piriformis muscle syndrome?.mp,kw. | 9 |
| 127 | polyarthralgia*.mp. | 9 |
| 128 | polymyalgia*.mp. | 206 |
| 129 | polyneuropath???.mp,kw. | 1337 |
| 130 | poly-neuropath???.mp,kw. | 13 |
| 131 | polyradiculitis.mp. | 2 |
| 132 | polyradiculoneuropath???.mp. | 233 |
| 133 | polyradiculopathies.mp. | 0 |
| 134 | polyradiculopathy.mp. | 17 |
| 135 | postherpetic.mp,kw. | 850 |
| 136 | post-herpetic.mp,kw. | 308 |
| 137 | radicular pain*.mp. | 601 |
| 138 | radiculitis.mp. | 62 |
| 139 | radiculopathies.mp. | 25 |
| 140 | radiculopathy.mp. | 1243 |
| 141 | sciatica.mp. | 886 |
| 142 | shingles.mp,kw. | 122 |
| 143 | trigeminal neuralgi*.mp. | 405 |
| 144 | or/25-143 [ Neuropathic Pain & Related Terms - EMTree & MeSH; December 31 2019 ] | 46436 |
| 145 | 24 and 144 | 100 |
| 146 | limit 145 to english language | 54 |
| 147 | remove duplicates from 146 | 53 |

# CDSR

Cochrane Database of Systematic Reviews 2005 to Present

| **#** | **Searches** | **Results** |
| --- | --- | --- |
| 1 | "hyperbaric?? oxygen*".ti,ab. | 30 |
| 2 | high pressure chamber?.ti,ab. | 0 |
| 3 | high pressure oxygen*.ti,ab. | 0 |
| 4 | high tension o2.ti,ab. | 0 |
| 5 | high tension oxygen*.ti,ab. | 0 |
| 6 | hyperbaric environment?.ti,ab. | 0 |
| 7 | hyperbaric room?.ti,ab. | 0 |
| 8 | hyperbaric unit?.ti,ab. | 0 |
| 9 | (hyperbaric? adj1 O2).ti,ab. | 0 |
| 10 | (hyperbaric? adj1 oxygen*).ti,ab. | 30 |
| 11 | (hyperbaric? adj2 chamber?).ti,ab. | 1 |
| 12 | (hyperbaric? adj2 medical*).ti,ab. | 1 |
| 13 | (hyperbaric? adj2 medicine).ti,ab. | 7 |
| 14 | (hyperbaric? adj2 therap*).ti,ab. | 25 |
| 15 | positive pressure chamber?.ti,ab. | 0 |
| 16 | positive pressure room?.ti,ab. | 0 |
| 17 | hbo.ti,ab. | 6 |
| 18 | hbo2.ti,ab. | 0 |
| 19 | hb-o2.ti,ab. | 0 |
| 20 | hbot.ti,ab. | 20 |
| 21 | or/1-20 [ Hyperbaric Oxygen Therapy & Related Terms ] | 31 |
| 22 | (central adj2 pain*).ti,ab. | 8 |
| 23 | (chemo* induced adj3 pain*).ti,ab. | 2 |
| 24 | (chemo-induced adj3 pain*).ti,ab. | 0 |
| 25 | (chemotherapy-induced adj3 pain*).ti,ab. | 2 |
| 26 | (deafferentation adj2 pain*).ti,ab. | 0 |
| 27 | (dysa?sthetic adj2 pain*).ti,ab. | 0 |
| 28 | (entrap* adj3 syndrom*).ti,ab. | 1 |
| 29 | (maladapt* adj2 pain*).ti,ab. | 1 |
| 30 | (mal-adapt* adj2 pain*).ti,ab. | 0 |
| 31 | (mononeurit* adj1 multiple*).ti,ab. | 0 |
| 32 | (mono-neurit* adj1 multiple*).ti,ab. | 0 |
| 33 | (Morton* adj1 neuroma*).ti,ab. | 1 |
| 34 | (nerve? adj12 pals???).ti,ab. | 10 |
| 35 | (nerve? adj2 damag*).ti,ab. | 30 |
| 36 | (nerve? adj2 injur*).ti,ab. | 15 |
| 37 | (nerve? adj2 sensitiv*).ti,ab. | 0 |
| 38 | (nerve? adj3 entrap*).ti,ab. | 1 |
| 39 | (neural adj2 damag*).ti,ab. | 0 |
| 40 | (neural adj2 injur*).ti,ab. | 0 |
| 41 | (neural adj3 entrap*).ti,ab. | 0 |
| 42 | (neural adj3 sensitiv*).ti,ab. | 0 |
| 43 | (neuro* adj2 pain*).ti,ab. | 69 |
| 44 | (neuro* adj2 sensitiv*).ti,ab. | 1 |
| 45 | (peripheral adj2 nerve? adj2 injur*).ti,ab. | 2 |
| 46 | (peripheral* adj1 mononeurit*).ti,ab. | 0 |
| 47 | (peripheral* adj1 mono-neurit*).ti,ab. | 0 |
| 48 | (peripheral* adj1 neurit*).ti,ab. | 0 |
| 49 | (peripheral* adj1 polyneurit*).ti,ab. | 0 |
| 50 | (peripheral* adj1 poly-neurit*).ti,ab. | 0 |
| 51 | (phantom limb? adj2 pain*).ti,ab. | 2 |
| 52 | (postamputat* adj2 pain*).ti,ab. | 1 |
| 53 | (post-amputat* adj2 pain*).ti,ab. | 0 |
| 54 | (postherpetic adj3 pain*).ti,ab. | 5 |
| 55 | (post-herpetic adj3 pain*).ti,ab. | 1 |
| 56 | (poststroke adj2 pain*).ti,ab. | 0 |
| 57 | (post-stroke adj2 pain*).ti,ab. | 5 |
| 58 | (pudendal adj2 entrap*).ti,ab. | 0 |
| 59 | (radiation* induced adj3 pain*).ti,ab. | 0 |
| 60 | (radiation-induced adj3 pain*).ti,ab. | 0 |
| 61 | (radiotherapy-induced adj3 pain*).ti,ab. | 0 |
| 62 | (somatosensory and pain*).ti,ab. | 3 |
| 63 | (surgery-induced adj3 pain*).ti,ab. | 0 |
| 64 | (surgically-induced adj3 pain*).ti,ab. | 0 |
| 65 | (trauma* adj3 nerve? trunk?).ti,ab. | 0 |
| 66 | allodynia*.ti,ab. | 2 |
| 67 | allodynic*.ti,ab. | 0 |
| 68 | arthralgia*.ti,ab. | 12 |
| 69 | arthrodynia*.ti,ab. | 0 |
| 70 | causalgi??.ti,ab. | 0 |
| 71 | cephalalgia?.ti,ab. | 4 |
| 72 | cephalodynia*.ti,ab. | 0 |
| 73 | cervicalgia*.ti,ab. | 0 |
| 74 | cervicodynia*.ti,ab. | 0 |
| 75 | cranialgia*.ti,ab. | 0 |
| 76 | dorsalgia*.ti,ab. | 0 |
| 77 | dysaesthesia*.ti,ab. | 1 |
| 78 | dysaesthesic*.ti,ab. | 0 |
| 79 | dysesthesia*.ti,ab. | 0 |
| 80 | dysesthetic*.ti,ab. | 0 |
| 81 | Herpes Zoster*.ti,ab. | 8 |
| 82 | hyper?esthesi*.ti,ab. | 3 |
| 83 | hyperalgesia*.ti,ab. | 3 |
| 84 | hyperpathia*.ti,ab. | 0 |
| 85 | hypo?esthesi*.ti,ab. | 1 |
| 86 | mononeuropath???.ti,ab. | 0 |
| 87 | mono-neuropath???.ti,ab. | 0 |
| 88 | multiple sclerosis.ti,ab. | 86 |
| 89 | myodynia*.ti,ab. | 0 |
| 90 | nerve root disorder?.ti,ab. | 0 |
| 91 | neuralgi*.ti,ab. | 34 |
| 92 | neuritis.ti,ab. | 3 |
| 93 | neuropathic.ti,ab. | 74 |
| 94 | neuropathies.ti,ab. | 13 |
| 95 | neuropathy.ti,ab. | 77 |
| 96 | nonnociceptive.ti,ab. | 0 |
| 97 | non-nociceptive.ti,ab. | 0 |
| 98 | numbness.ti,ab. | 9 |
| 99 | paraesthesia*.ti,ab. | 14 |
| 100 | paresthesia*.ti,ab. | 3 |
| 101 | perineuritis.ti,ab. | 0 |
| 102 | piriformis muscle syndrome?.ti,ab. | 0 |
| 103 | polyarthralgia*.ti,ab. | 0 |
| 104 | polymyalgia*.ti,ab. | 3 |
| 105 | polyneuropath???.ti,ab. | 13 |
| 106 | poly-neuropath???.ti,ab. | 0 |
| 107 | polyradiculitis.ti,ab. | 0 |
| 108 | polyradiculoneuropath???.ti,ab. | 6 |
| 109 | polyradiculopathies.ti,ab. | 0 |
| 110 | polyradiculopathy.ti,ab. | 0 |
| 111 | postherpetic.ti,ab. | 24 |
| 112 | post-herpetic.ti,ab. | 2 |
| 113 | radicular pain*.ti,ab. | 2 |
| 114 | radiculitis.ti,ab. | 0 |
| 115 | radiculopathies.ti,ab. | 1 |
| 116 | radiculopathy.ti,ab. | 14 |
| 117 | sciatica.ti,ab. | 13 |
| 118 | shingles.ti,ab. | 1 |
| 119 | trigeminal neuralgi*.ti,ab. | 8 |
| 120 | or/22-119 | 333 |
| 121 | 21 and 120 | 2 |
| 122 | limit 121 to full systematic reviews | 2 |

# Web of Science

**Web of Science Core Collection: Citation Indexes**

Science Citation Index Expanded (SCI-EXPANDED) --1900-present

Social Sciences Citation Index (SSCI) --1900-present

Arts & Humanities Citation Index (A&HCI) --1975-present

Conference Proceedings Citation Index- Science (CPCI-S) --1990-present

Conference Proceedings Citation Index- Social Science & Humanities (CPCI-SSH) --1990-present

Book Citation Index– Science (BKCI-S) --2005-present

Book Citation Index– Social Sciences & Humanities (BKCI-SSH) --2005-present

Emerging Sources Citation Index (ESCI) --2005-present

Data last updated: 2020-03-21

| # 4 | 497 | #2 AND #1  **Refined by:** [excluding] **DOCUMENT TYPES:** ( BOOK CHAPTER OR MEETING ABSTRACT )  Indexes=SCI-EXPANDED, SSCI, A&HCI, ESCI Timespan=All years |
| --- | --- | --- |
| # 3 | 534 | #2 AND #1  Indexes=SCI-EXPANDED, SSCI, A&HCI, ESCI Timespan=All years |
| # 2 | 388,879 | (TS=(Neuralgia OR Mononeuropathies OR Polyneuropathies OR "Nerve Compression Syndromes" OR ("Central Nervous System" AND "Wounds and Injuries") OR ("Central Nervous System" AND Pain*) OR "Central Nervous System injur*" OR "Complex Regional Pain Syndromes" OR "Peripheral Nerve Injuries" OR "Peripheral Nervous System Diseases" OR ("Peripheral Nervous System" AND "Wounds and Injuries") OR ("Peripheral Nervous System" AND Pain) OR "Peripheral Nervous System injur*" OR Causalgia OR "Piriformis Muscle Syndrome" OR "Reflex Sympathetic Dystrophy" OR (central NEAR/2 pain*) OR ("chemo* induced" NEAR/3 pain*) OR (chemo-induced NEAR/3 pain*) OR (chemotherapy-induced NEAR/3 pain*) OR (deafferentation NEAR/2 pain*) OR (dysa*sthetic NEAR/2 pain*) OR (entrap* NEAR/3 syndrom*) OR (maladapt* NEAR/2 pain*) OR (mal-adapt* NEAR/2 pain*) OR (mononeurit* NEAR/1 multiple*) OR (mono-neurit* NEAR/1 multiple*) OR (Morton* NEAR/1 neuroma*) OR (nerve* NEAR/12 pals*) OR (nerve* NEAR/2 damag*) OR (nerve* NEAR/2 injur*) OR (nerve* NEAR/2 sensitiv*) OR (nerve* NEAR/3 entrap*) OR (neural NEAR/2 damag*) OR (neural NEAR/2 injur*) OR (neural NEAR/3 entrap*) OR (neural NEAR/3 sensitiv*) OR (neuro* NEAR/2 pain*) OR (neuro* NEAR/2 sensitiv*) OR (peripheral NEAR/2 nerve* NEAR/2 injur*) OR (peripheral* NEAR/1 mononeurit*) OR (peripheral* NEAR/1 mono-neurit*) OR (peripheral* NEAR/1 neurit*) OR (peripheral* NEAR/1 polyneurit*) OR (peripheral* NEAR/1 poly-neurit*) OR ("phantom limb*" NEAR/2 pain*) OR (postamputat* NEAR/2 pain*) OR (post-amputat* NEAR/2 pain*) OR (postherpetic NEAR/3 pain*) OR (post-herpetic NEAR/3 pain*) OR (poststroke NEAR/2 pain*) OR (post-stroke NEAR/2 pain*) OR (pudendal NEAR/2 entrap*) OR ("radiation* induced" NEAR/3 pain*) OR (radiation-induced NEAR/3 pain*) OR (radiotherapy-induced NEAR/3 pain*) OR (somatosensory AND pain*) OR (surgery-induced NEAR/3 pain*) OR (surgically-induced NEAR/3 pain*) OR (trauma* NEAR/3 "nerve* trunk*") OR allodynia* OR allodynic* OR arthralgia* OR arthrodynia* OR causalgi* OR cephalalgia* OR cephalodynia* OR cervicalgia* OR cervicodynia* OR cranialgia* OR dorsalgia* OR dysaesthesia* OR dysaesthesic* OR dysesthesia* OR dysesthetic* OR "Herpes Zoster*" OR hyper*esthesi* OR hyperalgesia* OR hyperpathia* OR hypo*esthesi* OR mononeuropath* OR mono-neuropath* OR "multiple sclerosis" OR myodynia* OR "nerve root disorder*" OR neuralgi* OR neuritis OR neuropathic OR neuropathies OR neuropathy OR nonnociceptive OR non-nociceptive OR numbness OR paraesthesia* OR paresthesia* OR perineuritis OR "piriformis muscle syndrome*" OR polyarthralgia* OR polymyalgia* OR polyneuropath* OR poly-neuropath* OR polyradiculitis OR polyradiculoneuropath* OR polyradiculopathies OR polyradiculopathy OR postherpetic OR post-herpetic OR "radicular pain*" OR radiculitis OR radiculopathies OR radiculopathy OR sciatica OR shingles OR "trigeminal neuralgi*" )) *AND* **LANGUAGE:** (English)  Indexes=SCI-EXPANDED, SSCI, A&HCI, ESCI Timespan=All years |
| # 1 | 14,529 | (TS=("Hyperbaric Oxygenation" OR "hyperbaric* oxygen*" OR "high pressure chamber*" OR "high pressure oxygen*" OR "high tension o2" OR "high tension oxygen*" OR "hyperbaric environment*" OR "hyperbaric room*" OR "hyperbaric unit*" OR (hyperbaric* NEAR/1 O2) OR (hyperbaric* NEAR/1 oxygen*) OR (hyperbaric* NEAR/2 chamber*) OR (hyperbaric* NEAR/2 medical*) OR (hyperbaric* NEAR/2 medicine) OR (hyperbaric* NEAR/2 therap*) OR "positive pressure chamber*" OR "positive pressure room*" OR hbo OR hbo2 OR hb-o2 OR hbot )) *AND* **LANGUAGE:** (English)  Indexes=SCI-EXPANDED, SSCI, A&HCI, ESCI Timespan=All years |

# Scopus

348 document results

( INDEXTERMS ( *"Hyperbaric Oxygenation"* )  OR  TITLE-ABS-KEY ( *"hyperbaric* oxygen*"* )  OR  TITLE-ABS-KEY ( *"high pressure chamber*"* )  OR  TITLE-ABS-KEY ( *"high pressure oxygen*"* )  OR  TITLE-ABS-KEY ( *"high tension o2"* )  OR  TITLE-ABS-KEY ( *"high tension oxygen*"* )  OR  TITLE-ABS-KEY ( *"hyperbaric environment*"* )  OR  TITLE-ABS-KEY ( *"hyperbaric room*"* )  OR  TITLE-ABS-KEY ( *"hyperbaric unit*"* )  OR  ( *"hyperbaric*"*  W/1  *"O2"* )  OR  ( *"hyperbaric*"*  W/1  *"oxygen*"* )  OR  ( *"hyperbaric*"*  W/2  *"chamber*"* )  OR  ( *"hyperbaric*"*  W/2  *"medical*"* )  OR  ( *"hyperbaric*"*  W/2  *"medicine"* )  OR  ( *"hyperbaric*"*  W/2  *"therap*"* )  OR  TITLE-ABS-KEY ( *"positive pressure chamber*"* )  OR  TITLE-ABS-KEY ( *"positive pressure room*"* )  OR  TITLE-ABS-KEY ( *"hbo"* )  OR  TITLE-ABS-KEY ( *"hbo2"* )  OR  TITLE-ABS-KEY ( *"hb-o2"* )  OR  TITLE-ABS-KEY ( *"hbot"* ) )  AND  ( INDEXTERMS ( *"Neuralgia"* )  OR  INDEXTERMS ( *"Mononeuropathies"* )  OR  INDEXTERMS ( *"Polyneuropathies"* )  OR  INDEXTERMS ( *"Nerve Compression Syndromes"* )  OR  ( INDEXTERMS ( *"Central Nervous System"* )  AND  INDEXTERMS ( *"Wounds and Injuries"* ) )  OR  ( INDEXTERMS ( *"Central Nervous System"* )  AND  INDEXTERMS ( *"Pain"* ) )  OR  ( INDEXTERMS ( *"Central Nervous System"* )  *"in [Injuries]"* )  OR  INDEXTERMS ( *"Complex Regional Pain Syndromes"* )  OR  INDEXTERMS ( *"Peripheral Nerve Injuries"* )  OR  INDEXTERMS ( *"Peripheral Nervous System Diseases"* )  OR  ( INDEXTERMS ( *"Peripheral Nervous System"* )  AND  INDEXTERMS ( *"Wounds and Injuries"* ) )  OR  ( INDEXTERMS ( *"Peripheral Nervous System"* )  AND  INDEXTERMS ( *"Pain"* ) )  OR  INDEXTERMS ( *"Peripheral Nervous System"* )  *"in"*  OR  INDEXTERMS ( *"Causalgia"* )  OR  INDEXTERMS ( *"Piriformis Muscle Syndrome"* )  OR  INDEXTERMS ( *"Reflex Sympathetic Dystrophy"* )  OR  ( *"central"*  W/2  *"pain*"* )  OR  ( *"chemo* induced"*  W/3  *"pain*"* )  OR  ( *"chemo-induced"*  W/3  *"pain*"* )  OR  ( *"chemotherapy-induced"*  W/3  *"pain*"* )  OR  ( *"deafferentation"*  W/2  *"pain*"* )  OR  ( *"dysa*sthetic"*  W/2  *"pain*"* )  OR  ( *"entrap*"*  W/3  *"syndrom*"* )  OR  ( *"maladapt*"*  W/2  *"pain*"* )  OR  ( *"mal-adapt*"*  W/2  *"pain*"* )  OR  ( *"mononeurit*"*  W/1  *"multiple*"* )  OR  ( *"mono-neurit*"*  W/1  *"multiple*"* )  OR  ( *"Morton*"*  W/1  *"neuroma*"* )  OR  ( *"nerve*"*  W/12  *"pals*"* )  OR  ( *"nerve*"*  W/2  *"damag*"* )  OR  ( *"nerve*"*  W/2  *"injur*"* )  OR  ( *"nerve*"*  W/2  *"sensitiv*"* )  OR  ( *"nerve*"*  W/3  *"entrap*"* )  OR  ( *"neural"*  W/2  *"damag*"* )  OR  ( *"neural"*  W/2  *"injur*"* )  OR  ( *"neural"*  W/3  *"entrap*"* )  OR  ( *"neural"*  W/3  *"sensitiv*"* )  OR  ( *"neuro*"*  W/2  *"pain*"* )  OR  ( *"neuro*"*  W/2  *"sensitiv*"* )  OR  ( *"peripheral"*  W/2  *"nerve*"*  W/2  *"injur*"* )  OR  ( *"peripheral*"*  W/1  *"mononeurit*"* )  OR  ( *"peripheral*"*  W/1  *"mono-neurit*"* )  OR  ( *"peripheral*"*  W/1  *"neurit*"* )  OR  ( *"peripheral*"*  W/1  *"polyneurit*"* )  OR  ( *"peripheral*"*  W/1  *"poly-neurit*"* )  OR  ( *"phantom limb*"*  W/2  *"pain*"* )  OR  ( *"postamputat*"*  W/2  *"pain*"* )  OR  ( *"post-amputat*"*  W/2  *"pain*"* )  OR  ( *"postherpetic"*  W/3  *"pain*"* )  OR  ( *"post-herpetic"*  W/3  *"pain*"* )  OR  ( *"poststroke"*  W/2  *"pain*"* )  OR  ( *"post-stroke"*  W/2  *"pain*"* )  OR  ( *"pudendal"*  W/2  *"entrap*"* )  OR  ( *"radiation* induced"*  W/3  *"pain*"* )  OR  ( *"radiation-induced"*  W/3  *"pain*"* )  OR  ( *"radiotherapy-induced"*  W/3  *"pain*"* )  OR  ( *"somatosensory"*  AND  *"pain*"* )  OR  ( *"surgery-induced"*  W/3  *"pain*"* )  OR  ( *"surgically-induced"*  W/3  *"pain*"* )  OR  ( *"trauma*"*  W/3  *"nerve* trunk*"* )  OR  TITLE-ABS-KEY ( *"allodynia*"* )  OR  TITLE-ABS-KEY ( *"allodynic*"* )  OR  TITLE-ABS-KEY ( *"arthralgia*"* )  OR  TITLE-ABS-KEY ( *"arthrodynia*"* )  OR  TITLE-ABS-KEY ( *"causalgi*"* )  OR  TITLE-ABS-KEY ( *"cephalalgia*"* )  OR  TITLE-ABS-KEY ( *"cephalodynia*"* )  OR  TITLE-ABS-KEY ( *"cervicalgia*"* )  OR  TITLE-ABS-KEY ( *"cervicodynia*"* )  OR  TITLE-ABS-KEY ( *"cranialgia*"* )  OR  TITLE-ABS-KEY ( *"dorsalgia*"* )  OR  TITLE-ABS-KEY ( *"dysaesthesia*"* )  OR  TITLE-ABS-KEY ( *"dysaesthesic*"* )  OR  TITLE-ABS-KEY ( *"dysesthesia*"* )  OR  TITLE-ABS-KEY ( *"dysesthetic*"* )  OR  TITLE-ABS-KEY ( *"Herpes Zoster*"* )  OR  TITLE-ABS-KEY ( *"hyper*esthesi*"* )  OR  TITLE-ABS-KEY ( *"hyperalgesia*"* )  OR  TITLE-ABS-KEY ( *"hyperpathia*"* )  OR  TITLE-ABS-KEY ( *"hypo*esthesi*"* )  OR  TITLE-ABS-KEY ( *"mononeuropath*"* )  OR  TITLE-ABS-KEY ( *"mono-neuropath*"* )  OR  TITLE-ABS-KEY ( *"multiple sclerosis"* )  OR  TITLE-ABS-KEY ( *"myodynia*"* )  OR  TITLE-ABS-KEY ( *"nerve root disorder*"* )  OR  TITLE-ABS-KEY ( *"neuralgi*"* )  OR  TITLE-ABS-KEY ( *"neuritis"* )  OR  TITLE-ABS-KEY ( *"neuropathic"* )  OR  TITLE-ABS-KEY ( *"neuropathies"* )  OR  TITLE-ABS-KEY ( *"neuropathy"* )  OR  TITLE-ABS-KEY ( *"nonnociceptive"* )  OR  TITLE-ABS-KEY ( *"non-nociceptive"* )  OR  TITLE-ABS-KEY ( *"numbness"* )  OR  TITLE-ABS-KEY ( *"paraesthesia*"* )  OR  TITLE-ABS-KEY ( *"paresthesia*"* )  OR  TITLE-ABS-KEY ( *"perineuritis"* )  OR  TITLE-ABS-KEY ( *"piriformis muscle syndrome*"* )  OR  TITLE-ABS-KEY ( *"polyarthralgia*"* )  OR  TITLE-ABS-KEY ( *"polymyalgia*"* )  OR  TITLE-ABS-KEY ( *"polyneuropath*"* )  OR  TITLE-ABS-KEY ( *"poly-neuropath*"* )  OR  TITLE-ABS-KEY ( *"polyradiculitis"* )  OR  TITLE-ABS-KEY ( *"polyradiculoneuropath*"* )  OR  TITLE-ABS-KEY ( *"polyradiculopathies"* )  OR  TITLE-ABS-KEY ( *"polyradiculopathy"* )  OR  TITLE-ABS-KEY ( *"postherpetic"* )  OR  TITLE-ABS-KEY ( *"post-herpetic"* )  OR  TITLE-ABS-KEY ( *"radicular pain*"* )  OR  TITLE-ABS-KEY ( *"radiculitis"* )  OR  TITLE-ABS-KEY ( *"radiculopathies"* )  OR  TITLE-ABS-KEY ( *"radiculopathy"* )  OR  TITLE-ABS-KEY ( *"sciatica"* )  OR  TITLE-ABS-KEY ( *"shingles"* )  OR  TITLE-ABS-KEY ( *"trigeminal neuralgi*"* ) )  AND  ( LIMIT-TO ( LANGUAGE ,  *"English"* ) )  AND  ( EXCLUDE ( DOCTYPE ,  *"ch"* ) )

# ClinicalTrials.Gov

25 Studies found for: **pain OR neuralgia OR neuropathic OR neuropathy | Active, not recruiting, Completed, Suspended, Terminated, Withdrawn, Unknown status Studies | hyperbaric oxygen OR HBO OR HBOT OR Hyperbaric Chamber**

<https://clinicaltrials.gov/ct2/results/details?cond=hyperbaric+oxygen+OR+HBO+OR+HBOT+OR+Hyperbaric+Chamber&term=pain+OR+neuralgia+OR+neuropathic+OR+neuropathy&cntry=&state=&city=&dist=&Search=Search&recrs=d&recrs=e&recrs=g&recrs=h&recrs=i&recrs=m>

# WHO ICTRP


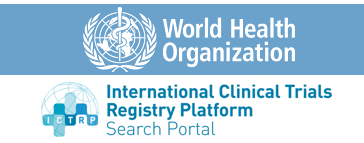


11 records for 11 trials found for: pain AND hyperbaric oxygen

# ProQuest Dissertations & Theses Global

"hyperbaric oxygen" AND ("neuropathic pain" OR neuralgia OR neuralgias OR neuropathy)

- Additional limits - Manuscript type: Doctoral dissertations; Language: English

322 results
